# The urinary microbiome: the next frontier of bacterial ecology

**DOI:** 10.1128/jb.00105-25

**Published:** 2025-07-24

**Authors:** Seth A. Reasoner, Jamisha Francis, Maria Hadjifrangiskou

**Affiliations:** 1 Division of Molecular Pathogenesis, Department of Pathology, Microbiology & Immunology, Vanderbilt University Medical Center, Nashville, Tennessee, USA; 2 Department of Urology, Vanderbilt University Medical Center, Nashville, Tennessee, USA; 3 Vanderbilt Institute for Infection, Immunology & Inflammation (VI4), Vanderbilt University Medical Center, Nashville, Tennessee, USA

**Keywords:** microbiome, urinary microbiome, urinary tract, urobiome

## Abstract

The human urinary tract, once presumed to be sterile, has emerged as a new frontier of microbial ecology. Recent advancements in high-throughput sequencing technologies have revealed the complexity and diversity of microbial communities that reside within the urinary tract. This mini-review discusses the prominent bacteria identified in the urinary microbiome and their correlations with various urologic conditions. This review serves to summarize the current state of urobiome research and chart a path for ongoing discovery. Additionally, we address the methodological challenges in urinary microbiome research, emphasizing the need for standardization in study protocols and the refinement of bioinformatics tools. We highlight that although differences in urobiome composition have been described for various urologic diseases. Similarly, the pathophysiologic source and consequences of those differences remain uncertain. We outline the steps to move urobiome research from descriptive to mechanistic studies, emphasizing rigorous study design, integrating multi-omics approaches, and developing robust model systems for experimental investigation. Finally, we outline critical questions for future investigation aimed at elucidating the intricate connections between the urinary microbiome and host health.

## A WALK THROUGH UROBIOME HISTORY

**FIG 1:**
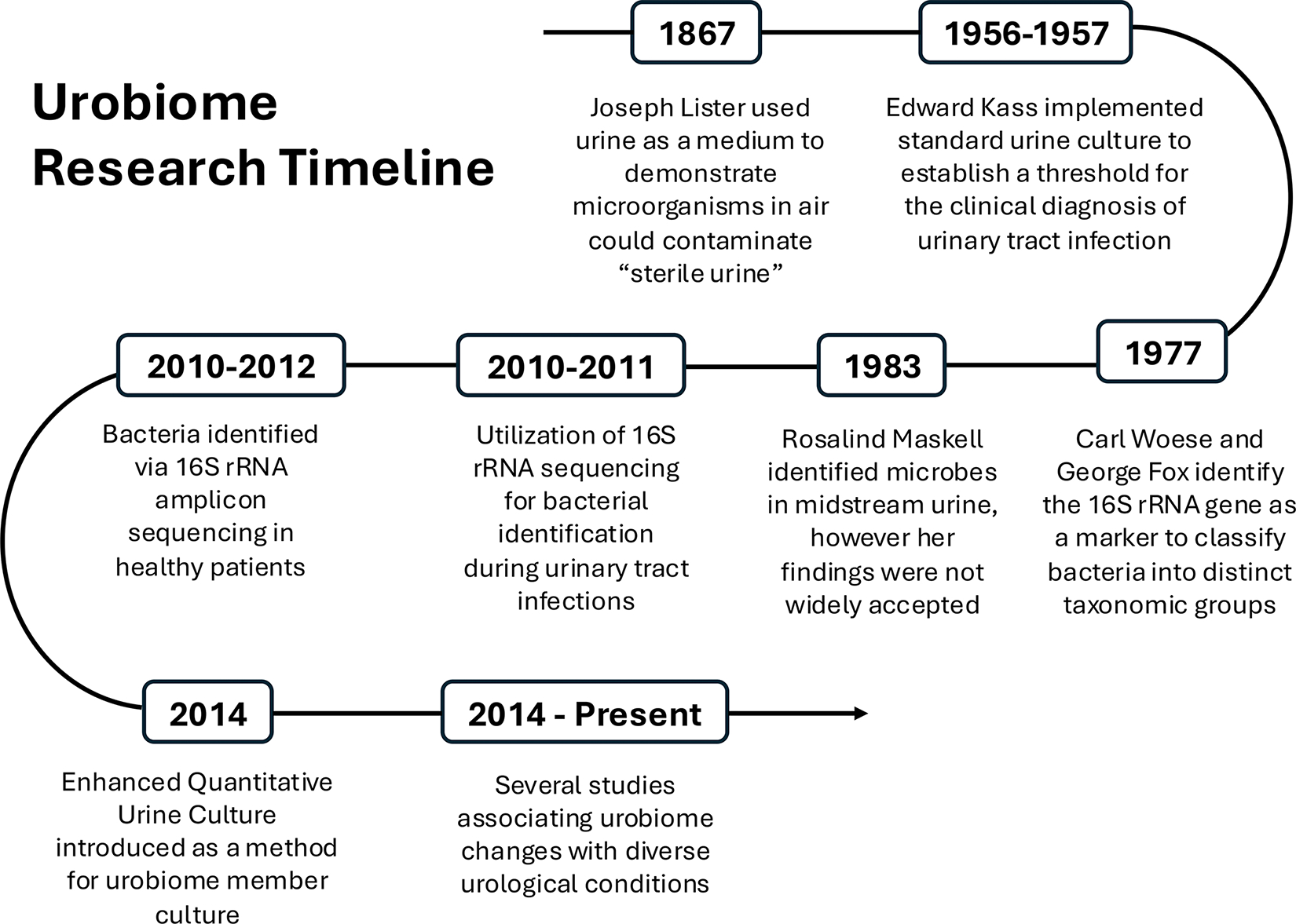
Chronology of urobiome discovery: a timeline outlining significant events that have contributed to the discovery of the urobiome and advancement of urobiome research.

Microbiological experimentation with human urine dates back to the late 1800s (Fig. 1). Joseph Lister, the pioneer of surgical antisepsis, used urine as a medium to demonstrate that microorganisms in the air could contaminate otherwise “sterile” urine (1, 2). In fact, Lister boiled the urine prior to these experiments, likely killing any native bacteria in the urine prior to initiating these experiments. In 1881, William Roberts published “On the Occurrence of Micro-Organisms in Fresh Urine,” concluding that fresh and healthy urine was free of bacteria (3). Roberts, who was a contemporary of Lister, undertook systematic experimentation to document the decomposition and alkalization of urine, processes now known to be mediated by microbes (3). He observed urine turbidity and microorganisms via microscopy. Roberts concluded that healthy urine was sterile and that the observed microorganisms originated from contamination after urine left the bladder, either from the skin and genitals or the air. With turbidity of the urine as the main method for detecting microbial growth, these early experiments lacked the sensitivity to detect slow-growing or fastidious microbes within the urine. These early conclusions ultimately halted the quest for the discovery of microbes within the healthy urinary tract.

In the 1950s, Edward Kass defined the difference between contamination and infection in the urinary tract (4–6). In his study, it was determined that urine from 95% of patients with symptoms of urinary tract infection (UTI) contained >10^5^ colony-forming units (CFU) per milliliter. Kass concluded, “a count of 10^5^ bacteria or more per mL of urine has been designated arbitrarily as the dividing line between true bacteriuria and contamination.” Based partly on this notion, the standard urine culture (SUC) was developed as the gold standard of urinary tract infection (UTI) diagnosis to identify potential UTI pathogens; SUC remains widely used to this day. The SUC protocol involves plating small volumes of urine (e.g., 1 μL) on 5% sheep blood agar and MacConkey agar, incubation in atmospheric conditions at 37°C for 24 h and evaluation of growth by medical technology personnel (4). These growth conditions are adequate for the growth of typical UTI culprits like *Escherichia, Klebsiella, and Proteus* but fail to identify other more fastidious microorganisms or strict anaerobes. As a result, many of the microbial inhabitants of the bladder go unidentified and unrecognized.

Beginning in 1967, Rosalind Maskell initiated her studies, experimenting with culture-based studies of SUC-negative urine samples and bladder biopsies (7–10). After decades of work, Maskell concluded that mid-stream urine samples do indeed contain bacteria, but they are unculturable by standard laboratory approaches (8, 11). However, her results did not gain widespread acceptance. In fact, the presence of cultured bacteria from voided urine samples not known to cause UTIs was dismissed as contaminants from the surrounding skin or genital microbes. In the early 2010s, the application of next-generation sequencing approaches and advanced culture techniques conclusively overturned the dogma that urine is sterile (11–20). Simultaneous sampling of skin or urethral swabs and urine demonstrated that bacteria were distinct between urine and skin or urethral samples. Although the use of 16S rRNA amplicon sequencing was routinely used for bacterial colony identification, the application of 16S rRNA to urine samples had not yet been explored (21). Since then, the confirmation and discovery of microbes in the urinary tract have been supported by a combination of 16S rRNA gene sequencing and expanded quantitative urine culture (EQUC) (18, 22–29). EQUC involves plating a larger volume of urine than SUC (100 μL vs 1 μL) on more nutritionally supportive media and incubating the cultures for longer in both aerobic and anaerobic environments. Many urine samples that do not exhibit growth by SUC show growth with EQUC. EQUC increases the EQUC has been instrumental in culturing viable bacteria—particularly more fastidious organisms—from urine samples. In the years since the widespread investigation of the urinary microbiome, dozens of studies have combined amplicon sequencing and culture methodologies to probe the microbial composition of urine specimens (30–43). Thus, the term urobiome was coined, referring to the microbiota of the urinary tract.

## CHALLENGES IN STUDYING THE UROBIOME

### General microbiome challenges exacerbated by the low biomass of the urobiome

The primary challenge of studying the urobiome is its relatively low microbial biomass (44), which is considerably lower than that found in more densely populated niches like the gut. Compared with the microbial composition of the gut, the urobiome has less than 10^5^ colony units per milliliter of urine, whereas the gut has 10^11^ bacteria per gram of feces (45, 46). This low bacterial load decreases the margin of error for introducing external contamination. Contamination introduced during the processing of urine samples has the potential to obscure the true signal from the low biomass of the sample. Additionally, low sample biomass complicates the DNA extraction process, which must be optimized for adequate extraction of DNA from low-abundance species. Differences in bacterial cell wall and membrane structures can increase the variability of DNA recovery from different isolation methods (47–49). Successful sequencing is heavily reliant on the efficient recovery of DNA from the sample. Some DNA extraction protocols may inadvertently amplify certain species over others or fail to extract DNA from microorganisms that are present in low abundance, leading to inconsistent results across studies. This highlights another challenge in the relatively nascent field of urobiome research: there is currently no universally accepted methodology for sampling, processing, or analyzing urinary microbiome data. Variability in extraction protocols, different 16S rRNA regions amplified, and differences in sequencing analysis across different studies can make it difficult to compare results or replicate findings (50). Additionally, there is a lack of sampling controls across urobiome studies that may lead to the reporting of contaminants as potential urobiome members, which can lead to incorrect interpretation of results. The contamination of urine samples poses a significant threat to data interpretation and may lead to overestimation of the bladder microbial community (51). The challenges of low biomass samples are not unique to urobiome research; similar challenges face tumor microbiome and breast milk microbiome research, for example (52–55). The use of cross-disciplinary techniques, sampling controls, and bioinformatic approaches from other low-biomass microbiome research endeavors can advance the reproducibility of urobiome research. For example, the biomass of urine has been estimated using EQUC. The limited media conditions included in EQUC cannot be expected to culture the diverse bacteria potentially present in the urinary tract. Thus, the biomass of urine remains uncertain. The use of precise techniques such as bacterial flow cytometry or quantitative PCR (qPCR) for 16S rRNA copy number would improve the precision of urine biomass estimates (56).

### Challenges with urine collection and processing

Another crucial challenge lies in sample collection and the mitigation of environmental contaminants. The method of urine collection can directly impact the accuracy and reliability of the microbial data obtained. There are multiple ways in which urine is collected, including midstream clean catch (“voided urine”), transurethral catheterization or suprapubic aspiration (57–62). Each method of urine collection has its advantages and disadvantages for urobiome research and influences the interpretation of the microbes identified (63–66). “Clean-catch” voided urine is the most accessible sample type, as it can be collected by the patient directly and not a healthcare provider. However, this poses the potential for contamination by vaginal, skin, or perianal flora (61). How carefully patients perform periurethral sterilization before providing a “clean-catch” sample is also highly variable. As a result, some have argued that voided urine should be referred to as a “urogenital sample” (67–70). Nonetheless, we argue that this represents the most representative snapshot of how the patient normally voids and the urinary tract’s microbial landscape at the time of collection. Moreover, if the skin or genital microbes are in close enough proximity introduced into voided urine, those microbes are likely to be in constant transit into the genitourinary tract and may contribute to genitourinary microbial ecology. As the least invasive method of urine collection, voided urine offers the lowest barrier to enrolling subjects and improving cohort sizes in urobiome studies. Transurethral catheterization involves placing a catheter into the bladder via the urethra. Sterilization of the periurethral area prior to catheterization decreases the potential for skin contaminants to be introduced into urine, and urine is drained directly from the bladder without encountering the urethral tissue. Nonetheless, transurethral catheterization is an uncomfortable procedure for patients, thus limiting its routine utility in collecting urine. During suprapubic aspiration, a needle is inserted through the skin of the lower abdomen, and urine is aspirated directly from the bladder. This is arguably the most stringent way to isolate microbes found inside the bladder, but it is the most invasive of the sampling methodologies. A skin swab of the lower abdomen may be used to exclude skin contamination from a suprapubic aspirate (18). Urine collected by either transurethral catheterization or suprapubic aspiration has lower microbial biomass, lower alpha diversity, and different microbial community composition compared with voided urine (18, 33, 63).

Sampling controls can be tailored to the particular study and method of sample collection. The inclusion of a periurethral or urethral swab as a control can attempt to differentiate skin, urethral, and genital microbes from microbes derived from voided urine (17, 67, 71, 72). Negative controls should include sampling blanks (e.g., sterile swabs or collection tubes exposed to the sampling environment), extraction blanks (reagent-only tubes processed alongside samples), and library preparation blanks to detect potential contamination introduced during DNA amplification and sequencing. Positive controls, such as mock microbial communities of known composition and abundance, help benchmark the performance of the sequencing pipeline, whereas spiked-in internal standards can aid in quantification and normalization (36, 55). Environmental controls—such as air exposure controls or surface swabs—are useful for identifying site-specific background contamination (20). Finally, technical replicates at the DNA extraction and sequencing stages provide insight into reproducibility and batch effects (73, 74).

Existing taxonomic databases are derived mainly from work in the gut microbiome and may not be optimized for classifying microbes from the urinary tract. Although databases for microbial identification are continually being updated, there is still a lack of a comprehensive reference database for urinary tract-associated microorganisms. This limitation makes it difficult to fully characterize the microbial communities in the urinary tract, as some less studied or novel species are not included in existing databases (75–77).

Below, we provide a synopsis of the most recent research elucidating the healthy urinary microbiomes and differences observed in urologic diseases. We compare sample sizes, sampling controls, and sequencing methodologies and propose an analysis framework going forward. Finally, we introduce bacterial species that have been consistently identified across urobiome studies. Further study of these species is warranted to dissect the role of these species in urinary tract health and disease. Next, we discuss the interconnections between the urinary, intestinal, and vaginal microbiomes.

## NEIGHBORING MICROBIAL COMMUNITIES: CONNECTIONS AND DISTINGUISHING FEATURES OF THE URINARY, INTESTINAL, AND VAGINAL MICROBIOMES

The urinary microbiome exists at the confluence of the skin, genital, and intestinal microbiomes. The unique nutrient pools and host immune pressures would intuitively alter each niche’s resident microbiota. Still, given the significantly higher biomass in the vaginal and intestinal microbiomes, many historically assumed that the urinary microbiome is solely an artifact of cross-contamination by these neighboring microbiomes. Elegant work from several groups demonstrated distinct bacterial taxa and functional roles between the urobiome and these “interconnected” microbiomes (19, 78, 79). In this section, we highlight the overlap between bacterial taxa between these neighboring microbiomes and key considerations for interpreting the urobiome in the context of the human microbiome at large.

As noted above, sample collection methods can influence the bacteria identified and interpretation of those results (Table 1). In studies where paired samples were obtained from the urinary tract and vaginal and/or intestinal tracts, the overlap of vaginal and urine samples is greater than the overlap with intestinal samples (19, 80–82). Recently, in a large study comprising urine, fecal, saliva, and vaginal samples, urine and vaginal samples showed the most similarity but were still able to be discriminated from one another (80). The discrepancies between samples were observed at both the bacterial phyla and genera levels. For example, at the phyla level, Actinobacteria are generally enriched in the urine relative to fecal samples, whereas Bacteroidetes are present in higher abundance in the intestinal tract (80). At the genus level, *Lactobacillus* (phyla Firmicutes) are more abundant in the urinary tract, whereas *Clostridium* and *Faecalibacterium* (also, phyla Firmicutes) are more abundant in the intestinal tract.

In women with urinary incontinence, the *Lactobacillus* genera occupied an average relative abundance of 53% of urine samples and 64% of vaginal samples, highlighting the significant overlap between these niches (78). Other genera—*Gardnerella*, *Prevotella*, and *Ureaplasma*—also showed high correlation coefficients between vaginal and urine specimens. In the same study, the presence of the genera *Tepidomonas* and *Flavobacterium* differentiated urine specimens from vaginal samples (78). More recently, identical strains of *Lactobacillus* have been described within the same subject’s vaginal and urine samples (83). Although similar strains of *Lactobacillus* may inhabit both the bladder and the vagina, their metabolism in each location is likely different. For example, the vagina is composed of glycogen which *Lactobacilli* metabolize to maintain a strictly acidic pH. In contrast, urine is relatively carbohydrate-depleted and can have a variable pH range, including both acidic and alkaline pHs. The differences in the metabolism of *Lactobacilli* in the bladder compared with the vagina remain unexplored.

To further illustrate the interconnection of neighboring microbiomes with the urobiome, we discuss the example of the urethral microbiome. Although the urethra is a part of the urinary tract, the urethral microbiome serves to demonstrate the nuances of interpreting urobiome results in light of the sample collection technique and their connection to neighboring microbiomes. We would anticipate that urethral microbes would be present in voided urine specimens, accumulated within urine as it exits the bladder, yet absent in catheter samples that transverse the urethra to sample urine. Moreover, as the most distal portion of the urinary tract, the urethra is most exposed to neighboring microbiomes, such as the vaginal microbiome. Consistent with this expectation, the microbial biomass of voided urine specimens is higher than the biomass of specimens obtained by catheter (18, 66). Similarly, paired urethral swabs and voided urine samples can differentiate microbes that originate in the urethra vs the bladder (29, 66, 84, 85). For example, the genera *Porphyromonas* and *Veillonella* (obligate anaerobes) appear to originate from the urethra in males (20, 29, 66). In contrast, *Dialister* and *Propionibacterium* (microaerophiles) appear to be derived from the bladder rather than the urethra (29). This highlights how urobiome results must be interpreted in light of (i) how the sample was collected and (ii) what neighboring microbiomes the sample may have been exposed to, that is, results from voided urine samples should only be compared with other voided samples and not specimens obtained via catheterization.

In summary, we emphasize that the urinary, vaginal, and intestinal niches are anatomically proximal. Indeed, we often observe similar taxa across these niches. These sites are likely in constant exchange of microbial members, with some of those exchanges being more persistent than others. Going forward, a key bioinformatic outlook is the longitudinal tracking of specific bacterial strains between interconnected microbiomes. Discriminating taxa that are uniquely abundant in one anatomic site offer excellent starting points for *in vitro* experimental validation to understand the unique metabolic features employed in each niche.

## HEALTHY UROBIOME: FREQUENTLY IDENTIFIED BACTERIAL MEMBERS

Several studies have probed the male and female urinary microbiomes in healthy subjects with no urologic comorbidities (Table 2). Notably, however, most of the “healthy subjects” within urobiome studies have been comparator groups when studying the urinary microbiomes of patients with different urologic diseases (44). Thus, studies dedicated to the “healthy” urobiome are scarce (30, 31, 70, 79). Several studies have begun elucidating urobiome development beginning in childhood, illustrating the presence of bacteria in the urinary tract as early as infancy (17, 20, 44, 86).

**FIG 2:**
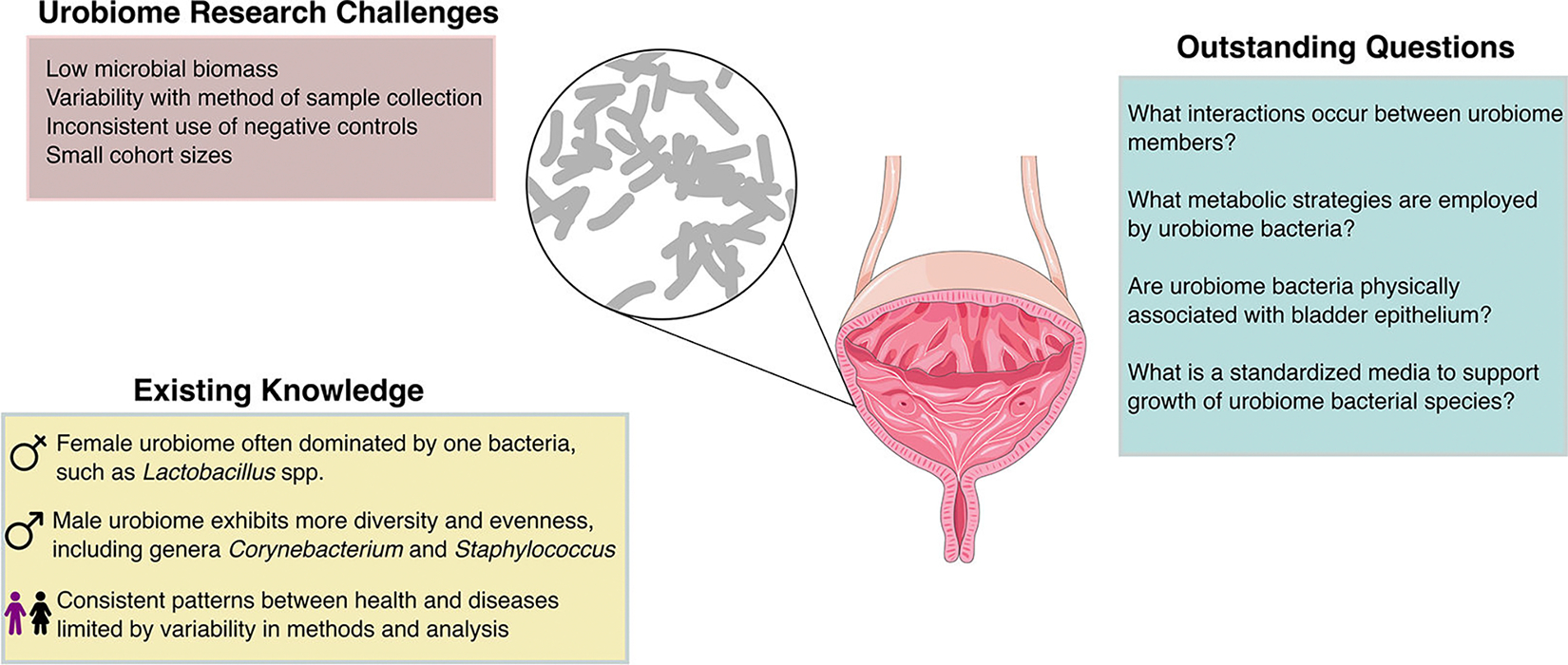
Schematic representing key concepts from this review. Figure designed in graphics editor Inkscape using open-source vector graphics from bioicons.com and bioart.niaid.nih.gov.

From the existing studies, the evidence indicates that there are distinct differences between the male and female urobiomes and across age groups (Fig. 2). The male urobiome has been reported to have higher alpha diversity compared with females; this increased diversity is most pronounced in males below the age of 60 years (70). The adult female urinary microbiome is characterized by a high abundance of various *Lactobacillus* and *Gardnerella* species (36, 41, 70, 93, 94). The high abundance of *Lactobacillus* among many female urobiomes may explain the decreased alpha diversity relative to males. The abundance of *Lactobacillus* increases in the female urinary microbiome during puberty and then decreases following menopause (17, 31, 70, 85). On the other hand, the male urobiome is characterized by a high abundance of *Corynebacterium*, *Propionibacterium*, and *Staphylococcus* species (30, 33, 70, 89). Other frequently detected genera in healthy subjects include *Aerococcus*, *Atopobium*, *Actinotignum*, *Bacteroides*, *Bifidobacterium*, *Peptoniphilus*, *Prevotella*, *Streptococcus*, *Ureaplasma*, *Varibaculum,* and *Veillonella* (45, 95). Urinary pathogens, including the genera *Escherichia, Pseudomonas, Klebsiella, Proteus, Staphylococcus*, and *Enterococcus,* are frequently detected in urine samples from patients without UTI (70). This suggests that urinary pathogens can be frequently present at levels undetectable by SUC. This observation underscores the complexity of interpreting microbial presence in the urinary tract, challenging the binary classification of urinary pathogens as either infectious or benign. This phenomenon is best exemplified by asymptomatic bacteriuria (ASB) in which urinary pathogens are present at high titer; however, the patient does not exhibit symptoms. Urinary pathogens and UTIs have been reviewed extensively elsewhere and will not be discussed further (96–99).

To ascribe an overall label to an individual’s urobiome composition, the concept of an “urotype” was developed (44). Urotypes are determined based on the specimen’s dominant bacterial species or genera composition. Urotypes represent a similar classification system to vaginal community state types (CSTs) and intestinal enterotypes (100, 101). For example, intestinal enterotypes include Bacteroides-dominated, Prevotella-dominated, and Ruminococcus-dominated community classifications (102). Between different studies, different urotypes have been described (44, 70, 73, 80, 89, 103). For example, urotypes include communities dominated by: *Lactobacillus*, *Gardnerella*, *Escherichia*, *Prevotella*, *Streptococcus*, *Staphylococcus*, and a mixed microbial community type. A *Lactobacillus* urotype appears to be the most common among pre-menopausal women, whereas an *Escherichia* or *Enterococcus* urotype is more common among older individuals (31, 74). In a small longitudinal study, some subjects had stable urotypes (often *Lactobacillus* predominance), whereas other subjects had less stable urotypes often with changes in relation to sexual activity and/or menstruation (71). Although describing urotypes has become increasingly common within urobiome research, what constitutes a “healthy” urotype or patient factors that influence urotype remain uncertain.

## WHO’S WHO IN THE UROBIOME

The majority of urobiome studies have been descriptive, given the nascent nature of the field. We argue that the next frontier lies in mechanistic studies to elucidate how microbial activities in the bladder influence diverse urologic conditions including urinary tract infections. Although there is an overlap in taxa between the urobiome and neighboring microbiomes, we would anticipate different functional roles for urobiome members because of the unique metabolic nutrient pool in urine. We briefly highlight frequently identified urobiome members that warrant mechanistic study to determine their role in the urobiome. We have selected these genera given the frequency at which they are identified by both culture and sequencing in urobiome studies. Moreover, these genera have known or proposed functional roles in microbial ecology, urinary health, and infection susceptibility.

### *Lactobacillus* spp.

*Lactobacillus* species are predominant members of the urinary microbiome, particularly in women (39, 41, 74). Common urinary *Lactobacillus* species include *Lactobacillus crispatus*, *Lactobacillus gasseri*, *Lactobacillus jensenii*, and *Lactobacillus iners* (19, 77)*. Lactobacilli* often comprise >50% of the relative abundance of bacteria from female urine samples (41, 104). *Lactobacilli* have been primarily studied in relation to UTI susceptibility; *Lactobacilli* are inversely correlated with the risk of UTI, especially in postmenopausal women (36, 39, 104). Although *Lactobacilli* are known to produce lactic acid, which lowers the surrounding pH, the effect of *Lactobacilli* on urine pH is uncertain. Urine pH has a variable range (pH 4–8), and no studies have explored whether urinary pH correlates with *Lactobacilli* abundance (105). Additionally, *Lactobacillus* species produce bacteriocins, antimicrobial peptides that directly inhibit other bacterial community members (106). Preliminary evidence also suggests that *Lactobacilli* modulate host bladder immune function to resist colonization by urinary pathogens (104, 107). Given their central role in urobiome ecology, a focused study of *Lactobacilli* within the urobiome will uncover their metabolism employed in urine and interactions with other urobiome bacterial members.

### Prevotella

The genus *Prevotella* comprises gram-negative anaerobic bacteria commonly found in various human microbiomes, including the oral cavity, gut, and vagina. Numerous studies indicate that *Prevotella* is among the most prevalent and abundant genera in the urinary microbiomes of healthy individuals (63, 70, 80). Although *Prevotella* is known for metabolizing dietary fibers in the intestinal microbiome and mucin in the vaginal microbiome (108, 109), *Prevotella* metabolism in the urinary tract has not been studied. Based on *Prevotella* metabolism in other niches, we speculate that *Prevotella* may metabolize glycoproteins from the urinary epithelium or amino acids, which are abundant in urine. In other niches, *Prevotella* is a prodigious producer of short-chain fatty acids (SCFAs), which regulate immune functions and promote epithelial cell integrity (110). Interrogation of the metabolism and immune interplay of *Prevotella* in the urobiome is a worthwhile endeavor.

### Actinotignum

The genus *Actinotignum* is increasingly recognized within the urinary microbiome. The *Actinotignum* genus consists of three known species *Actinotignum schaalii*, *Actinotignum urinale*, and *Actinotignum sanguinis* (111–114). Although *Actinotignum* spp. are repeatedly identified in healthy urine samples, they have also been associated with opportunistic infections, particularly in older males and patients with underlying health conditions (115–119). A study analyzing 86 clinical *Actinotignum* infection isolates found that 43% were from blood cultures and 7% from urine samples, with UTIs clinically presumed to be the source of most bacteremia cases (120). However, simultaneous standard urine cultures rarely yielded positive results for *Actinotignum*, likely due to its fastidious growth requirements and the limitations of typical laboratory methods in detecting this organism. As a result, *Actinotignum* may go undetected as a cause of some UTIs (121, 122). Genomic analysis of *Actinotignum* isolates suggests that carbohydrate metabolism would occur glycolytically or via the pentose phosphate and the Entner-Doudoroff pathways (123). Furthermore, *Actinotignum* isolates encode multiple siderophore receptors but not the genes for siderophore biosynthesis, potentially indicating a role for the inter-species exchange of siderophores within the urobiome (20). These observations offer intriguing mechanistic targets to understand the metabolism of *Actinotignum* spp. in the urinary tract.

### Aerococcus

Like *Actinotignum*, the genus *Aerococcus* is frequently detected in urine samples, although generally not a dominant urobiome member (18, 24). *Aerococcus* spp., and particularly the species *A. urinae,* are now acknowledged as potential pathogens in UTIs, especially among elderly individuals with urinary tract abnormalities (124). Studies have isolated *A. urinae* from urine samples from subjects of all ages (20, 74, 125, 126). Preliminary studies have demonstrated the presence of *Aerococcus* in catheter biofilms and the ability to form aggregates in liquid culture (126–128). A mouse model of *Aerococcus* UTI displayed a tropism for the kidneys even following clearance of bacteria in the urine (129). Furthermore, *Aerococcus* is illustrative of the challenges of taxonomic classification of urobiome bacterial species. Whole genome sequencing of urinary *Aerococcus* isolates has revealed multiple previously uncharacterized species (130).

Both the genera *Actinotignum* and *Aerococcus* exemplify that frequently detected urobiome members across patient categories may contribute to UTIs in specific patient groups, complicating the commensal-pathogen continuum.

## UROBIOME IN UROLOGIC DISEASES

The aforementioned bacterial taxa and other bacterial members of the urobiome will be discussed in the context of specific diseases in which they have been identified (Table 2). We highlight what can be gleaned about bacterial ecology from different urological disease states.

### Benign prostatic hyperplasia

Benign prostatic hyperplasia (BPH) is a common condition in older men that is characterized by the non-cancerous enlargement of the prostate gland (131). Prostate enlargement impairs urine emptying. BPH is thought to contribute to the elevated rates of UTIs among older men, suggesting that a change in urobiome ecology may occur due to BPH and that these ecological changes may be associated with increased infection susceptibility to non-classical uropathogens like *Actinotignum* spp (119, 132).

The urobiome of patients with BPH has been associated with increased alpha diversity when compared with men without BPH (33, 34, 89, 133). The relative abundance of specific bacterial genera is increased in urine samples from BPH patients such as *Haemophilus*, *Staphylococcus*, and *Faecalibacterium* compared with control subjects (88). *Haemophilus* was also associated with increased prostate-specific antigen (PSA) levels, a laboratory metric of prostate enlargement (88). In 2024, Mariotti et al. reported an association between *Lactobacillus* and elevated PSA levels. These disparate results may be attributable to different urine sampling methodologies (voided vs. catheterized) between these studies (88, 90). BPH treatment to shrink prostate size with 5-alpha reductase inhibitors further modifies the urobiome with an increased abundance of *Corynebacterium* and *Anaerococcus* (90). Given the anatomic connection between the prostate and bladder, sampling of prostate tissue directly has shown promise for precisely defining the spatial localization of bacteria in the prostate vs. the bladder at large (134). Given the frequency of BPH among older men and its association with UTIs, future research should explore how urobiome ecological changes may be associated with infection susceptibility and tissue inflammation in BPH.

### Interstitial cystitis/bladder pain syndrome

Interstitial cystitis/bladder pain syndrome (IC/BPS) is a chronic urological condition that affects 4–12 million people in the United States (135–137). IC/BPS causes severe pelvic pain that is temporarily relieved by urination. Only a minority of patients have identifiable bladder inflammation, leading to the disease being considered a chronic pain condition rather than an inflammatory disease. The cause of IC/BPS remains uncertain and is likely multi-factorial, including psychosocial factors, epithelial abnormalities, and inappropriate immune activation (138). Because of the overlap in symptoms between IC/BPS and UTIs, whether bacteria contribute to IC/BPS has been a longstanding question. Many of the first urobiome studies investigated IC/BPS given the potential microbial contribution to disease etiology (139–146).

The urobiome of IC/BPS patients compared with healthy controls has consistently shown reduced alpha diversity (27, 147–149). However, the specific bacterial taxa contributing to this reduced alpha diversity varies across studies (150, 151). For example, there are conflicting results regarding the abundance of *Lactobacilli* in IC/BPS relative to healthy controls (27, 148, 152). Abernathy et al., Xu et al., and Zheng et al. reported reduced *Lactobacilli* relative to healthy controls (87, 147, 148). In contrast, Siddiqui et al. and Nickel et al. reported an increased abundance of *Lactobacilli* relative to healthy controls (27, 152). These studies varied in their sample collection technique (voided urine vs. catheterization) and methodologies for taxonomic determination (16S rRNA sequencing vs. mass spectrometric identification), limiting the ability to draw meaningful conclusions between studies (151). The abundance of *Lactobacillus* could also be impacted by the menopausal status of participants (153). The prevalence of urinary pathogens (*Escherichia*, *Klebsiella*, etc.) is low in IC/BPS, contradicting the historical assumption that IC/BPS may be an inflammatory response to typical urinary pathogens (154, 155).

Another endeavor has been to associate IC/BPS sub-types or symptom flares with the urobiome (145, 155). Nickel et al. demonstrated that there was no urobiome difference in women with Hunner’s lesions compared with non-Hunner’s lesion IC/BPS, the characteristic ulcerative inflammatory bladder lesion observed in a minority of patients (145). In contrast, men with Hunner’s lesion had an increased abundance of *Negativicoccus succinivorans*, *Porphyromonas somerae*, and *Mobiluncus curtisii* and a reduced abundance of *Corynebacterium renale*. In a separate study, a higher prevalence of fungi (*Candida* and *Saccharomyces*) was noted in women with IC/BPS symptom flares (155). Once again, despite some associational evidence, no conclusive evidence exists that the urobiome strongly influences symptom severity or flares in IC/BPS.

At present, IC/BPS is unanimously considered a non-infectious condition (156–160). That is, there is no “dysbiotic” urobiome signature that is universally identified in IC/BPS. Variable methodologies between IC/BPS urobiome studies have complicated the ability to draw comparisons across studies. Thus, whether specific members of the urobiome contribute to IC/BPS symptomatology remains an open question.

### Urinary incontinence and overactive bladder

Urinary incontinence and overactive bladder are similar urologic conditions that have been the focus of multiple urobiome studies (23, 37, 38, 41, 44, 103, 161–165). Urinary incontinence (UI) is a common condition characterized by the involuntary loss of urine, whereas overactive bladder (OAB) is characterized by frequent urge to urinate and can be associated with incontinence. The urobiome has been postulated as a modifying factor of symptoms in UI and OAB (44, 165).

The results from urobiome studies in UI and OAB exemplify the disparate results that can confound meaningful conclusions. That is, differences observed between patients with UI or OAB and healthy controls have been variable between studies. No associations have been reproducibly associated with UI and OAB or these diseases’ symptom severities. Price et al. reported that healthy controls tend to exhibit higher levels of *Lactobacillus* than UI patients (73). Conversely, Komesu et al. reported no difference in *Lactobacillus* abundance in UI vs. control patients, although this association is likely confounded by menopausal status (92, 94). Carnes et al. assessed the relationship between UI severity and urobiome community types (i.e., urotypes). Six distinct urobiome profiles were identified. Community 1, the *Lactobacillus* dominant community, displayed lower symptom severity when compared with Community 3, which displayed lower levels of *Lactobacillus* and was dominated by *Tepidimonas* and *Acidovorax* (41). Intriguingly, a mechanistic study of different bacterial species indicated that *E. coli* and *Gardnerella vaginalis* increased calcium influx and contraction in urothelial cells and myofibroblasts, cellular mechanisms that may underlie symptomology in UI and OAB (166). In contrast, *Lactobacillus* spp. did not induce calcium movement and cellular contraction (166). This study highlights how observations from sequencing experiments can be used to interrogate urobiome-mediated cellular mechanisms of urology disease.

### Urinary stone disease

Urinary stone disease or urolithiasis refers to solid crystalline concretions that form within the urinary system and can cause episodes of acute pain such as renal colic (167). The most common type of urinary stone disease is nephrolithiasis (kidney stones). The pathogenesis of urinary stone disease is a complex interplay of host genetic, metabolic, microbiologic, and dietary factors that culminate in an imbalance of the metabolites in urine. The connection between the urobiome and urinary stone disease is 2-fold: (i) certain types of bacteria may contribute to the formation of urinary stones via their metabolic products and (ii) stones create a scaffold for biofilm formation and predispose afflicted individuals to stone-associated infections. Bacteria associated with urolithiasis can be studied from urine or by direct culture or sequencing of stones themselves.

The most common type of urolith is calcium oxalate stones. The genus *Lactobacillus* has repeatedly been shown to be enriched in healthy controls compared with patients with calcium oxalate stones (168–170). In contrast, *Corynebacterium* is increased in urine samples from individuals with calcium oxalate stones (43, 91). For example, *Corynebacterium* is increased in urine collected from the kidney with a struvite stone compared to contralateral kidney without a stone (91). Urinary pathogens, particularly from the family *Enterobacteriaceae*, are also enriched in calcium oxalate stones (168, 171, 172). Whether urinary pathogens contribute to the metabolic development of calcium oxalate stones or opportunistically infect the stone architecture remains uncertain (171).

Struvite stones comprise a minority of kidney stones yet have the most direct bacterial contribution to stone formation. Struvite stones form as a result of a UTI caused by urease-producing bacteria (173). *Proteus, Klebsiella, Pseudomonas*, and *Corynebacterium* are urease-producing bacteria that are commonly associated with kidney stone formation. Urease-producing bacteria alkalinize the urine by producing ammonium; alkaline urine can lead to the precipitation of phosphate which forms the crystalline struvite stones (174). Although *Proteus*, *Klebsiella*, and *Pseudomonas* are known urinary pathogens, *Corynebacterium* spp. are frequently detected in the urinary microbiome in the absence of infection (80). For example, *Corynebacterium urealyticum* was shown to induce struvite stone formation when inoculated into the bladders of rats (175).

Among the urologic diseases that we discuss, urolithiasis has the most consistent urobiome findings across studies. *Lactobacillus* is consistently depleted in the urobiome from multiple types of urinary stones, whereas *Corynebacterium* is enriched in individuals with either calcium oxalate or struvite stones. In sum, although diet and host genetics are established risk factors for urolithiasis, the urobiome may play a role in modulating an individual’s risk of urinary stones.

## OUTLOOK OF UROBIOME RESEARCH

The developing field of urobiome research has transformed our understanding of the urinary tract from a sterile environment to a dynamic microbial ecosystem. Advances in sequencing technologies, particularly 16S rRNA amplicon sequencing, have revealed a diverse community of bacteria with potential implications for urinary health and disease. Although significant progress has been made in developing techniques for the urobiome, many questions remain regarding the functional roles of these bacteria, their interactions with the host, and their influence on conditions such as UTIs, BPH, incontinence, and other bladder disorders.

Urobiome research has the potential to transform urology diagnostics and treatments. For example, SUC remains the gold standard of UTI diagnosis decades after its development. As noted, SUC does not capture the diversity of the urinary tract. New molecular diagnostics are being developed to leverage urobiome research to understand imbalances in microbial communities that may indicate healthy vs. diseased states (176). Likewise, a deeper understanding of the microbial ecology of the urinary tract may advance more targeted urinary therapeutics. For example, although *Lactobacillus*-containing probiotics are frequently marketed and employed for UTI prevention, no studies have assessed whether those probiotics reach the urinary tract, stably colonize the bladder, and impact urobiome ecology (177, 178).

A challenge, which is not unique to urobiome research, is the inherent difficulty in establishing causal relationships between microbiome composition and disease states. Although differences in the urinary microbiome have been observed in conditions like BPH and interstitial cystitis, it is often unclear whether these changes are causal, coincidental, or a consequence of the disease. The ability to sequence microbial samples outpaces our ability to rigorously interpret and synthesize results across studies. That is, it is far easier to sequence 100 urine samples and identify associations with patient variables than it is to experimentally determine what those associations represent.

The next decade of urobiome research offers the opportunity to move beyond correlational cross-sectional studies and concentrate on mechanisms of bacterial metabolism and polymicrobial interactions in the urobiome. We offer our suggestions for establishing experimental models to test the mechanistic roles of urobiome members. We take inspiration from examples in the intestinal microbiome and cystic fibrosis respiratory microbiome fields, which have made great strides in translating sequencing observations to understand the functional role of individual microbial members in the community (179–181).

In contrast to the gastrointestinal microbiome, the urobiome harbors a smaller contingent of bacterial species. This fact should be harnessed as mechanistic research into the urobiome continues. Namely, fewer species present in an individual’s urobiome offer a unique advantage in modeling the polymicrobial interactions in the urobiome. Bacterial species should be selected based on consistent identification across urobiome studies by both culture and sequencing methods. Initial studies should prioritize bacterial species that can be readily grown in a laboratory setting and informed by those microbes’ characteristics in other settings (e.g., *Lactobacillus* urobiome research should be informed by its known characteristics in the vagina). Moreover, urine is arguably a more replicable media for laboratory studies than the gastrointestinal milieu. Urine is predominately composed of amino acids, salts, and nitrogenous waste (urea and creatinine), which is easier to replicate than gastrointestinal contents. These advantages that are inherent to the urobiome ecosystem can be leveraged to develop experimental models.

First, sequencing data should be mined to guide experimental approaches. Bioinformatic analysis of existing sequencing data should be used to identify patterns of bacterial co-occurrence and potential metabolic complementarity. These patterns would inform the design of microbial consortia—smaller groups of bacterial species that may interact functionally and ecologically in the urobiome. Simpler consortia of bacteria—often dyads or triads of bacterial species—allow the testing of polymicrobial interactions and causal relationships under controlled conditions. A similar approach, moving from sequencing data to microbial consortia, from cystic fibrosis microbiome research developed a four-species consortium for *in vitro* testing (180).

To test polymicrobial interactions of urobiome members, standardized and representative media must be used to mimic native conditions in the bladder. Laboratory recipes for artificial urine have been published (182–185). Artificial urine recipes have been designed for the study of urinary pathogens and not tailored to the growth requirements of more fastidious urobiome members. Although artificial urine recipes do not perfectly recapitulate the composition of human urine, these recipes offer a foundation for developing a reliable medium for polymicrobial co-culture experiments of urobiome members. For example, glycosaminoglycans from the bladder epithelium were recently shown to be associated with the abundance of particular urobiome members (186); however, artificial urine recipes do not contain glycosaminoglycans. Existing urea and creatinine-based artificial urine recipes must be further optimized to support the growth of fastidious urobiome members.

To conclude, we pose several questions to spur mechanistic research in the urobiome. What is the spatial localization of urobiome members, and how do they physically associate with urinary tract epithelium? What are the/custom tear similarities and differences between bacterial species isolated from the urine and isolated from the vagina or intestinal tract? Do urobiome members obtain nutrients from urine, or do they cross-feed each other? What are the metabolic programs used by urobiome members to grow in urine? What urobiome members are frequently co-occurring, and what are their metabolic interactions? In conclusion, future mechanistic research addressing such questions will identify biologically meaningful and/or clinically actionable insights from the urobiome.

## Figures and Tables

**TABLE 1:** Factors influencing variability in urobiome study results

Experimental aspect	Pitfall examples	Proposed solutions

Sample collection (voided urine, transurethral catheterization, or suprapubic aspiration)	Biomass differs between sample collection procedures and the likelihood of urethral/skin microbes within urine samples.	Compare studies with the same method of sample collection with consideration for the introduction of microbes from neighboring anatomic sites.
Sampling controls	Sampling controls are not collected or reported.	Consistent collection and reporting of sampling controls that are relevant for the study design and sample collection procedures.
DNA extraction	Different DNA extraction kits have different propensities for extracting DNA from particular bacterial species.	Incorporate mock microbial communities and negative controls to benchmark DNA extraction from different bacterial species.
Sequencing technology	Different regions of the 16S rRNA have variable resolution of taxonomic groups.	Compare studies in light of sequencing technology and biases in technologies.
Bioinformatic and statistical analysis	Inconsistencies in bioinformatic or statistical analyses confound generalizability of results.	Analyze samples consistently across studies and re-analyze publicly available data with uniform analytic processes.
Accessible data and code	Sequencing source data and bioinformatic code are not routinely publicly uploaded.	Upload sequencing data to public data repositories and bioinformatic code to promote transparency and reproducibility.

**TABLE 2:** Summary of study features and reported urobiome composition based on urological conditions, Specimen Type, Methodologies Employed in Studies, and Participant Demographics^a^

Urological condition	PMID#	Specimen source	Study technique	Number of participants disease vs. control	Notable microbes present in control subjects	Notable microbes present in diseased state

Healthy cohort	Siddiqui et al. (26): 22047020	Voided Urine	16SrRNA V1-V2 & V6 regions	eight healthy females	*Lactobacillus, Prevotella, Gardnerella, Peptoniphilus, Dialister, Finegoldia, Anaerococcus, Allisonella, Streptococcus,* and *Staphylococcus*	N/A^b^
	Qin et al. (70): 34489882	Voided Urine	16S rRNA V4 region	1,172 participants between the age of 45 and 86	*Prevotella, Streptococcus, Lactobacillus, Gardnerella, Escherichia-Shigella,* and *Veillonella*	N/a
	Nickel et al. (30) 35426787	Voided Urine	Plex-ID Kit and ESI-TOF-MS	Participants with IC/BPS (primarily females) and CP/CPPS (males), as well as asymptomatic control subjects	*Bifidobacterium, Staphylococcus, Lactobacillus, Corynebacterium, Propionibacterium*	N/A
	Reasoner et al. (20): 38040700	Transurethral Catheter	16S rRNA V4 region and enhanced urine culture	50 male infants	*Escherichia-Shigella, Prevotella, Nocardiopsis, Lacibacter, Staphylococcus,* and *Lactobacillus*	N/A
	Zou et al. (80): 39513726	Voided Urine	Metagenomic sequencing	1579 individuals	*Lactobacillus, Variovorax, Acinetobacter, Prevotella, Sphingobium*	N/A
Interstitial cystitis/bladder pain syndrome (IC/BPS)	Jacobs et al., 2020: 32265402	Transurethral Catheter	16S rRNA V4 region and EQUC	40 women with IC/PBS and 40 controls	*Lactobacillus, Facklamia, Staphylococcus, Gardnerella*	*Streptococcus, Actinomyces, Corynebacterium, Escherichia, Lactobacillus*
	Zheng et al. (87): 37076836	Voided Urine	16S rRNA V3-V4 regions	30 IC/BPS patients and 30 healthy controls	*Lactobacillus, Acinetobacter*	*Ralstonia*
Benign prostatic hyperplasia (BPH)	Lee et al. (88): 33858430	Voided urine	16S rRNA V3-V4 regions	77 men with BPH and 30 control participants without recent antibiotic use	N/A	*Lactobacillus, Staphylococcus, Bacillus, Faecalibacterium, Listeria, Enhydrobacter, Pseudomonas, Neisseria, Phascolarctobacterium, Dolosigranulum, Haemophilus, [Ruminococcus]torques, Bamesiella, Finegoldia, Prevotellaceae NK3B31 group*
	Bowie et al. (89): 38168244	Voided Urine	16S rRNA V4 region	500 randomly selected from Osteoporotic Fractures in Men (MrOS) cohort	*Corynebacterium, Staphylococcus, Streptococcus*	*Dialister, Actinotignum, Prevotella*
Mariotti et al. (90): 37961000	Transurethral Catheter	16S rRNAV1-V2 region	41 men with BPH undergoing transurethral resection of the prostate	N/A	*Corynebacterium, Lactobacillus, Variovorax, Staphylococcus, Cutibacterium*	
Nephrolithiasis (kidney stones)	Liu et al. (91): 33153435	Suprapubic aspirate	16S rRNA V3-V4 region and EQUC	219 samples	*Bifidobacterium, Pseudomonas*	*Sphingomonas, Pontibacter, Bifidobacterium, Prevotella, Corynebacterium*
	Dornbier et al. (43): 31240349	Transurethral Catheter	16S rRNA V4 region and EQUC	101 patients, 52 with stones	N/A	*Staphylococcus, Veillonella, Streptococcus, Corynebacterium, Haemophilus, Proteus, Lactobacillus, Bifidobacterium, Escherichia, Klebsiella*
	Hong et al. (51): 36153619	Renal pelvis urine	Type IIB Restriction-site Associated DNA sequencing for Microbiome	30 patients with unilateral stones	Non-stone side: *Prevotella, Lactobacillus, Pseudomonas*	Stone side: *Corynebacterium*
	Kachroo et al. (42): 35234986	Voided Urine	Metagenomic sequencing	five with ultrasound confirmed kidney stones and five with no history of kidney stones	*Lactobacillus*	*Pseudomonas, Burkholderia*
Overactive bladder and urinary incontinence (UI)	Hilt et al. (24): 24371246	Transurethral Catheter	16S rRNA V4 region, Standard Culture and EQUC	41 patients with overactive bladder and 24 controls	*Lactobacillus, Streptococcus, Staphylococcus, Micrococcus, Bifidobacterium, Enterococcus, Alloscardovia, Rothia, Neisseria*	*Corynebacterium, Actinomyces, Aerococcus, Actinobaculum, Arthrobacter, Facklamia, Oligello*
	Pearce et al. (44): 25006228	Transurethral Catheter	16S rRNA V4 regions and EQUC	EQUC for 45 UUI and 45 non- UUI samples and 16S rRNA gene sequencing for 36 UUI and 38 non-UUl samples	*Atopobium, Bifidobacterium, Corynebacterium, Finegoldia, Lachnospiraceae, Peptoniphilus, Rhoanobacter, Staphylococcus, Streptococcus*	*Actinobaculum, Actinomyces, Aerococcus, Anaerococcus, Campylobacter, Dialister, Facklamia, Faecalibacterium, Fastidiosipila, Gardnerella, Mobiluncus, Porphyromonas, Prevotella, Reuminococcaceae, Sneathia, Ureaplasma*
	Komesu et al. (92): 29909556	Transurethral Catheter	16SrRNAV4 - V6 regions	Multicenter study, 123 MUI and 84 controls	*Lactobacillus, Streptococcus, Staphylococcus*	*Gardnerella*
	Carnes et al. (41): 38937257	Transurethral Catheter	16S rRNA V4 - V6 regions and EQUC	126 of 480 with MUI	*Lactobacillus, Streptococcus, Staphylococcus*	*Prevotella, Tepidomonas, Escherichia, Acidovorax*

a EQUC—Enhanced quantitative urine culture; CP/CPPS—Chronic Prostatitis/Chronic Pelvic Pain Syndrome; ESI-TOF— Electrospray Ionization Time-of-Flight; UUI—urge urinary incontinence; MUI—mixed urinary incontinence.

b N/A, No notable associations were observed in the diseased state.

## References

[1] Ford WW. 1928. The bacteriological work of Joseph Lister. Sci Mon 26:70–75.

[2] Newsom SWB. 2003. Pioneers in infection control—Joseph Lister. J Hosp Infect 55:246–253. 10.1016/j.jhin.2003.08.001 14629967

[3] Roberts W 1881. On the occurrence of micro-organisms in fresh urine. Br Med J 2:623–625. 10.1136/bmj.2.1085.623 PMC2263882 20750001

[4] KASS EH. 1956. Asymptomatic infections of the urinary tract. Trans Assoc Am Physicians 69:56–64. 10.1016/s0022-5347(02)80328-7 13380946

[5] Kass EH. 1957. Bacteriuria and the diagnosis of infections of the urinary tract; with observations on the use of methionine as a urinary antiseptic. AMA Arch Intern Med 100:709–714. 10.1001/archinte.1957.00260110025004 13468815

[6] Khasriya R, Sathiananthamoorthy S, Ismail S, Kelsey M, Wilson M, Rohn JL, Malone-Lee J. 2013. Spectrum of bacterial colonization associated with urothelial cells from patients with chronic lower urinary tract symptoms. J Clin Microbiol 51:2054–2062. 10.1128/JCM.03314-12 23596238 PMC3697662

[7] Maskell R, Pead L, Sanderson RA. 1983. Fastidious bacteria and the urethral syndrome: a 2-year clinical and bacteriological study of 51 women. Lancet 2:1277–1280. 10.1016/s0140-6736(83)91152-2 6139621

[8] Maskell RM. 2010. The natural history of urinary tract infection in women. Med Hypotheses 74:802–806. 10.1016/j.mehy.2009.12.011 20064694

[9] Brumfitt W, Hamilton-Miller JM, Ludlam H, Gooding A. 1981. Lactobacilli do not cause frequency and dysuria syndrome. Lancet 2:393–396. 10.1016/s0140-6736(81)90833-3 6115160

[10] Wilkins EGL, Payne SR, Pead PJ, Moss ST, Maskell RM. 1989. Interstitial cystitis and the urethral syndrome: a possible answer. Br J Urol 64:39–44. 10.1111/j.1464-410X.1989.tb05519.x 2670041

[11] Brubaker L, Wolfe AJ. 2015. The new world of the urinary microbiota in women. Am J Obstet Gynecol 213:644–649. 10.1016/j.ajog.2015.05.032 26003055 PMC4876712

[12] Wolfe AJ, Brubaker L. 2020. Ur-ine old age: urinary microbiome of older community dwelling women. Cell Host Microbe 28:149–151. 10.1016/j.chom.2020.07.012 32791105

[13] Elsayed NS, Wolfe AJ, Burk RD. 2023. Urine microbiome in individuals with an impaired immune system. Front Cell Infect Microbiol 13:1308665. 10.3389/fcimb.2023.1308665 38274734 PMC10808152

[14] Joyce C, Halverson T, Gonzalez C, Brubaker L, Wolfe AJ. 2022. The urobiomes of adult women with various lower urinary tract symptoms status differ: a re-analysis. Front Cell Infect Microbiol 12:860408. 10.3389/fcimb.2022.860408 35755842 PMC9218574

[15] Halverson T, Mueller ER, Brubaker L, Wolfe AJ. 2023. Urobiome changes differ based on OAB treatment in adult females. Int Urogynecol J 34:1271–1277. 10.1007/s00192-022-05416-x 36422657

[16] Burnett LA, Hochstedler BR, Weldon K, Wolfe AJ, Brubaker L. 2021. Recurrent urinary tract infection: association of clinical profiles with urobiome composition in women. Neurourol Urodyn 40:1479–1489. 10.1002/nau.24707 34036621 PMC8298270

[17] Storm DW, Copp HL, Halverson TM, Du J, Juhr D, Wolfe AJ. 2022. A Child’s urine is not sterile: a pilot study evaluating the Pediatric Urinary Microbiome. J Pediatr Urol 18:383–392. 10.1016/j.jpurol.2022.02.025 35337731

[18] Wolfe AJ, Toh E, Shibata N, Rong R, Kenton K, Fitzgerald M, Mueller ER, Schreckenberger P, Dong Q, Nelson DE, Brubaker L. 2012. Evidence of uncultivated bacteria in the adult female bladder. J Clin Microbiol 50:1376–1383. 10.1128/JCM.05852-11 22278835 PMC3318548

[19] Thomas-White K, Forster SC, Kumar N, Van Kuiken M, Putonti C, Stares MD, Hilt EE, Price TK, Wolfe AJ, Lawley TD. 2018. Culturing of female bladder bacteria reveals an interconnected urogenital microbiota. Nat Commun 9:1557. 10.1038/s41467-018-03968-5 29674608 PMC5908796

[20] Reasoner SA, Flores V, Van Horn G, Morales G, Peard LM, Abelson B, Manuel C, Lee J, Baker B, Williams T, Schmitz JE, Clayton DB, Hadjifrangiskou M. 2023. Survey of the infant male urobiome and genomic analysis of *Actinotignum* spp. NPJ Biofilms Microbiomes 9:91. 10.1038/s41522-023-00457-6 38040700 PMC10692110

[21] Woese CR, Fox GE. 1977. Phylogenetic structure of the prokaryotic domain: the primary kingdoms. Proc Natl Acad Sci USA 74:5088–5090. 10.1073/pnas.74.11.5088 270744 PMC432104

[22] Price TK, Dune T, Hilt EE, Thomas-White KJ, Kliethermes S, Brincat C, Brubaker L, Wolfe AJ, Mueller ER, Schreckenberger PC. 2016. The clinical urine culture: enhanced techniques improve detection of clinically relevant microorganisms. J Clin Microbiol 54:1216–1222. 10.1128/JCM.00044-16 26962083 PMC4844725

[23] Karstens L, Asquith M, Davin S, Stauffer P, Fair D, Gregory WT, Rosenbaum JT, McWeeney SK, Nardos R. 2016. Does the urinary microbiome play a role in urgency urinary incontinence and its severity? Front Cell Infect Microbiol 6:78. 10.3389/fcimb.2016.00078 27512653 PMC4961701

[24] Hilt EE, McKinley K, Pearce MM, Rosenfeld AB, Zilliox MJ, Mueller ER, Brubaker L, Gai X, Wolfe AJ, Schreckenberger PC. 2014. Urine is not sterile: use of enhanced urine culture techniques to detect resident bacterial flora in the adult female bladder. J Clin Microbiol 52:871–876. 10.1128/JCM.02876-13 24371246 PMC3957746

[25] Fouts DE, Pieper R, Szpakowski S, Pohl H, Knoblach S, Suh M-J, Huang S-T, Ljungberg I, Sprague BM, Lucas SK, Torralba M, Nelson KE, Groah SL. 2012. Integrated next-generation sequencing of 16S rDNA and metaproteomics differentiate the healthy urine microbiome from asymptomatic bacteriuria in neuropathic bladder associated with spinal cord injury. J Transl Med 10:174. 10.1186/1479-5876-10-174 22929533 PMC3511201

[26] Siddiqui H, Nederbragt AJ, Lagesen K, Jeansson SL, Jakobsen KS. 2011. Assessing diversity of the female urine microbiota by high throughput sequencing of 16S rDNA amplicons. BMC Microbiol 11:244. 10.1186/1471-2180-11-244 22047020 PMC3228714

[27] Siddiqui H, Lagesen K, Nederbragt AJ, Jeansson SL, Jakobsen KS. 2012. Alterations of microbiota in urine from women with interstitial cystitis. BMC Microbiol 12:205. 10.1186/1471-2180-12-205 22974186 PMC3538702

[28] Nelson DE, Van Der Pol B, Dong Q, Revanna KV, Fan B, Easwaran S, Sodergren E, Weinstock GM, Diao L, Fortenberry JD. 2010. Characteristic male urine microbiomes associate with asymptomatic sexually transmitted infection. PLoS One 5:e14116. 10.1371/journal.pone.0014116 21124791 PMC2991352

[29] Dong Q, Nelson DE, Toh E, Diao L, Gao X, Fortenberry JD, Van Der Pol B. 2011. The microbial communities in male first catch urine are highly similar to those in paired urethral swab specimens. PLoS One 6:e19709. 10.1371/journal.pone.0019709 21603636 PMC3094389

[30] Nickel JC, Stephens A, Ackerman AL, Anger JT, Lai HH, Ehrlich GD. 2022. The healthy urinary microbiome in asymptomatic participants in the MAPP Network Study: relation to gender, age, and menopausal status. Can Urol Assoc J 16:E448–E454. 10.5489/cuaj.7775 35426787 PMC9484748

[31] Ammitzbøll N, Bau BPJ, Bundgaard-Nielsen C, Villadsen AB, Jensen AM, Leutscher PDC, Glavind K, Hagstrøm S, Arenholt LTS, Sørensen S. 2021. Pre- and postmenopausal women have different core urinary microbiota. Sci Rep 11:2212. 10.1038/s41598-021-81790-8 33500504 PMC7838182

[32] Curtiss N, Balachandran A, Krska L, Peppiatt-Wildman C, Wildman S, Duckett J. 2018. Age, menopausal status and the bladder microbiome. Eur J Obstet Gynecol Reprod Biol 228:126–129. 10.1016/j.ejogrb.2018.06.011 29936400

[33] Bajic P, Van Kuiken ME, Burge BK, Kirshenbaum EJ, Joyce CJ, Wolfe AJ, Branch JD, Bresler L, Farooq AV. 2020. Male bladder microbiome relates to lower urinary tract symptoms. Eur Urol Focus 6:376–382. 10.1016/j.euf.2018.08.001 30143471

[34] Shrestha E, White JR, Yu S-H, Kulac I, Ertunc O, De Marzo AM, Yegnasubramanian S, Mangold LA, Partin AW, Sfanos KS. 2018. Profiling the urinary microbiome in men with positive versus negative biopsies for prostate cancer. J Urol 199:161–171. 10.1016/j.juro.2017.08.001 28797714 PMC5937117

[35] Hurst R, Meader E, Gihawi A, Rallapalli G, Clark J, Kay GL, Webb M, Manley K, Curley H, Walker H, 2022. Microbiomes of urine and the prostate are linked to human prostate cancer risk groups. Eur Urol Oncol 5:412–419. 10.1016/j.euo.2022.03.006 35450835

[36] Neugent ML, Kumar A, Hulyalkar NV, Lutz KC, Nguyen VH, Fuentes JL, Zhang C, Nguyen A, Sharon BM, Kuprasertkul A, Arute AP, Ebrahimzadeh T, Natesan N, Xing C, Shulaev V, Li Q, Zimmern PE, Palmer KL, De Nisco NJ. 2022. Recurrent urinary tract infection and estrogen shape the taxonomic ecology and function of the postmenopausal urogenital microbiome. Cell Rep Med 3:100753. 10.1016/j.xcrm.2022.100753 36182683 PMC9588997

[37] Curtiss N, Balachandran A, Krska L, Peppiatt-Wildman C, Wildman S, Duckett J. 2017. A case controlled study examining the bladder microbiome in women with Overactive Bladder (OAB) and healthy controls. Eur J Obstet Gynecol Reprod Biol 214:31–35. 10.1016/j.ejogrb.2017.04.040 28463826

[38] Thomas-White K, Taege S, Limeira R, Brincat C, Joyce C, Hilt EE, Mac-Daniel L, Radek KA, Brubaker L, Mueller ER, Wolfe AJ. 2020. Vaginal estrogen therapy is associated with increased *Lactobacillus* in the urine of postmenopausal women with overactive bladder symptoms. Am J Obstet Gynecol 223:727. 10.1016/j.ajog.2020.08.006 PMC7609597 32791124

[39] Vaughan MH, Mao J, Karstens LA, Ma L, Amundsen CL, Schmader KE, Siddiqui NY. 2021. The urinary microbiome in postmenopausal women with recurrent urinary tract infections. J Urol 206:1222–1231. 10.1097/JU.0000000000001940 34181466 PMC8497440

[40] Richter HE, Carnes MU, Komesu YM, Lukacz ES, Arya L, Bradley M, Rogers RG, Sung VW, Siddiqui NY, Carper B, Mazloomdoost D, Dinwiddie D, Gantz MG. 2022. Association between the urogenital microbiome and surgical treatment response in women undergoing midurethral sling operation for mixed urinary incontinence. Am J Obstet Gynecol 226:93. 10.1016/j.ajog.2021.07.008 PMC8748268 34297969

[41] Carnes MU, Siddiqui NY, Karstens L, Gantz MG, Dinwiddie DL, Sung VW, Bradley M, Brubaker L, Ferrando CA, Mazloomdoost D, Richter HE, Rogers RG, Smith AL, Komesu YM, Eunice Kennedy Shriver National Institute of Child Health and Human Development Pelvic Floor Disorders Network. 2024. Urinary microbiome community types associated with urinary incontinence severity in women. Am J Obstet Gynecol 230:344. 10.1016/j.ajog.2023.10.036 PMC11211640 38937257

[42] Kachroo N, Lange D, Penniston KL, Stern J, Tasian G, Bajic P, Wolfe AJ, Suryavanshi M, Ticinesi A, Meschi T, Monga M, Miller AW. 2021. Meta-analysis of clinical microbiome studies in urolithiasis reveal age, stone composition, and study location as the predominant factors in urolithiasis-associated microbiome composition. mBio 12:e0200721. 10.1128/mBio.02007-21 34372696 PMC8406293

[43] Dornbier RA, Bajic P, Van Kuiken M, Jardaneh A, Lin H, Gao X, Knudsen B, Dong Q, Wolfe AJ, Schwaderer AL. 2020. The microbiome of calcium-based urinary stones. Urolithiasis 48:191–199. 10.1007/s00240-019-01146-w 31240349

[44] Pearce MM, Hilt EE, Rosenfeld AB, Zilliox MJ, Thomas-White K, Fok C, Kliethermes S, Schreckenberger PC, Brubaker L, Gai X, Wolfe AJ. 2014. The female urinary microbiome: a comparison of women with and without urgency urinary incontinence. mBio 5:e01283–14. 10.1128/mBio.01283-14 25006228 PMC4161260

[45] Perez-Carrasco V, Soriano-Lerma A, Soriano M, Gutiérrez-Fernández J, Garcia-Salcedo JA. 2021. Urinary microbiome: yin and yang of the urinary tract. Front Cell Infect Microbiol 11:617002. 10.3389/fcimb.2021.617002 34084752 PMC8167034

[46] Berg RD. 1996. The indigenous gastrointestinal microflora. Trends Microbiol 4:430–435. 10.1016/0966-842X(96)10057-3 8950812

[47] Krsek M, Wellington EMH. 1999. Comparison of different methods for the isolation and purification of total community DNA from soil. J Microbiol Methods 39:1–16. 10.1016/S0167-7012(99)00093-7 10579502

[48] Felczykowska A, Krajewska A, Zielińska S, Łoś JM. 2015. Sampling, metadata and DNA extraction - important steps in metagenomic studies. Acta Biochim Pol 62:151–160. 10.18388/abp.2014_916 25680373

[49] Carrigg C, Rice O, Kavanagh S, Collins G, O’Flaherty V. 2007. DNA extraction method affects microbial community profiles from soils and sediment. Appl Microbiol Biotechnol 77:955–964. 10.1007/s00253-007-1219-y 17960375

[50] Bukin YS, Galachyants YP, Morozov IV, Bukin SV, Zakharenko AS, Zemskaya TI. 2019. The effect of 16S rRNA region choice on bacterial community metabarcoding results. Sci Data 6:190007. 10.1038/sdata.2019.7 30720800 PMC6362892

[51] Kim JK, Song SH, Jung G, Song B, Hong SK. 2022. Possibilities and limitations of using low biomass samples for urologic disease and microbiome research. Prostate Int 10:169–180. 10.1016/j.prnil.2022.10.001 36570648 PMC9747588

[52] Ensuring rigour of low-biomass microbiome research. 2025. Nat Microbiol 10:261–262. 10.1038/s41564-025-01939-3 39905170

[53] Pust M-M, Rocha Castellanos DM, Rzasa K, Dame A, Pishchany G, Assawasirisin C, Liss A, Fernandez-Del Castillo C, Xavier RJ. 2024. Absence of a pancreatic microbiome in intraductal papillary mucinous neoplasm. Gut 73:1131–1141. 10.1136/gutjnl-2023-331012 38429112 PMC11187374

[54] Kennedy KM, de Goffau MC, Perez-Muñoz ME, Arrieta M-C, Bäckhed F, Bork P, Braun T, Bushman FD, Dore J, de Vos WM, 2023. Questioning the fetal microbiome illustrates pitfalls of low-biomass microbial studies. Nature 613:639–649. 10.1038/s41586-022-05546-8 36697862 PMC11333990

[55] Karstens L, Asquith M, Davin S, Fair D, Gregory WT, Wolfe AJ, Braun J, McWeeney S. 2019. Controlling for contaminants in low-biomass 16S rRNA gene sequencing experiments. mSystems 4:e00290–19. 10.1128/mSystems.00290-19 PMC6550369 31164452

[56] Doyle B, Reynolds GZM, Dvorak M, Maghini DG, Natarajan A, Bhatt AS. 2025. Absolute quantification of prokaryotes in the microbiome by 16S rRNA qPCR or ddPCR. Nat Protoc. 10.1038/s41596-025-01165-5 PMC13547822 40389632

[57] Barnes WF, Albers DD. 1978. Comparison of paired midstream voided and catheterized urine samples from female patients in a general hospital. J Okla State Med Assoc 71:82–84. 632958

[58] Dawborn JK, Plunkett PJ. 1963. The collection and assessment op mid-stream urine samples in the diagnosis of urinary tract infection in women. Med J Aust 1:540–543. 10.5694/j.1326-5377.1963.tb23261.x 14025566

[59] Bradbury SM. 1988. Collection of urine specimens in general practice: to clean or not to clean? J R Coll Gen Pract 38:363–365. 3256648 PMC1711498

[60] Lifshitz E, Kramer L. 2000. Outpatient urine culture: does collection technique matter? Arch Intern Med 160:2537–2540. 10.1001/archinte.160.16.2537 10979067

[61] LaRocco MT, Franek J, Leibach EK, Weissfeld AS, Kraft CS, Sautter RL, Baselski V, Rodahl D, Peterson EJ, Cornish NE. 2016. Effectiveness of preanalytic practices on contamination and diagnostic accuracy of urine cultures: a laboratory medicine best practices systematic review and meta-analysis. Clin Microbiol Rev 29:105–147. 10.1128/CMR.00030-15 26598386 PMC4771218

[62] Schneeberger C, van den Heuvel ER, Erwich JJHM, Stolk RP, Visser CE, Geerlings SE. 2013. Contamination rates of three urine-sampling methods to assess bacteriuria in pregnant women. Obstet Gynecol 121:299–305. 10.1097/AOG.0b013e31827e8cfe 23344279

[63] Pohl HG, Groah SL, Pérez-Losada M, Ljungberg I, Sprague BM, Chandal N, Caldovic L, Hsieh M. 2020. The urine microbiome of healthy men and women differs by urine collection method. Int Neurourol J 24:41–51. 10.5213/inj.1938244.122 32252185 PMC7136448

[64] Wolfe AJ, Brubaker L. 2019. Urobiome updates: advances in urinary microbiome research. Nat Rev Urol 16:73–74. 10.1038/s41585-018-0127-5 30510275 PMC6628711

[65] Brubaker L, Wolfe AJ. 2017. Microbiota in 2016: associating infection and incontinence with the female urinary microbiota. Nat Rev Urol 14:72–74. 10.1038/nrurol.2016.262 28050013 PMC5522000

[66] Hrbacek J, Morais D, Cermak P, Hanacek V, Zachoval R. 2021. Alpha-diversity and microbial community structure of the male urinary microbiota depend on urine sampling method. Sci Rep 11:23758. 10.1038/s41598-021-03292-x 34887510 PMC8660768

[67] Brubaker L, Gourdine J-PF, Siddiqui NY, Holland A, Halverson T, Limeria R, Pride D, Ackerman L, Forster CS, Jacobs KM, Thomas-White KJ, Putonti C, Dong Q, Weinstein M, Lukacz ES, Karstens L, Wolfe AJ. 2021. Forming consensus to advance urobiome research. mSystems 6:e0137120. 10.1128/mSystems.01371-20 34282932 PMC8409733

[68] Jeries LM, Sysoeva TA, Karstens L, Kelly MS. 2024. Synthesis of current pediatric urinary microbiome research. Front Pediatr 12:1396408. 10.3389/fped.2024.1396408 38957777 PMC11217333

[69] Karstens L, Asquith M, Caruso V, Rosenbaum JT, Fair DA, Braun J, Gregory WT, Nardos R, McWeeney SK. 2018. Community profiling of the urinary microbiota: considerations for low-biomass samples. Nat Rev Urol 15:735–749. 10.1038/s41585-018-0104-z 30315209 PMC6352978

[70] Qin J, Shi X, Xu J, Yuan S, Zheng B, Zhang E, Huang G, Li G, Jiang G, Gao S, Tian C, Guo R, Fu Z, Huang Q, Yang R, Zhang W, Li S, Wu S. 2021. Characterization of the genitourinary microbiome of 1,165 middle-aged and elderly healthy individuals. Front Microbiol 12:673969. 10.3389/fmicb.2021.673969 34489882 PMC8417382

[71] Price TK, Wolff B, Halverson T, Limeira R, Brubaker L, Dong Q, Mueller ER, Wolfe AJ. 2020. Temporal dynamics of the adult female lower urinary tract microbiota. mBio 11:e00475–20. 10.1128/mBio.00475-20 32317321 PMC7175091

[72] Stewart E, Hochstedler-Kramer BR, Khemmani M, Clark NM, Parada JP, Farooq A, Doshi C, Wolfe AJ, Albarillo FS. 2024. Characterizing the urobiome in geriatric males with chronic indwelling urinary catheters: an exploratory longitudinal study. Microbiol Spectr 12:e0094124. 10.1128/spectrum.00941-24 39387607 PMC11536997

[73] Price TK, Lin H, Gao X, Thomas-White KJ, Hilt EE, Mueller ER, Wolfe AJ, Dong Q, Brubaker L. 2020. Bladder bacterial diversity differs in continent and incontinent women: a cross-sectional study. Am J Obstet Gynecol 223:729. 10.1016/j.ajog.2020.04.033 PMC7609606 32380174

[74] Price TK, Hilt EE, Thomas-White K, Mueller ER, Wolfe AJ, Brubaker L. 2020. The urobiome of continent adult women: a cross-sectional study. BJOG 127:193–201. 10.1111/1471-0528.15920 31469215 PMC7197444

[75] McDonald D, Price MN, Goodrich J, Nawrocki EP, DeSantis TZ, Probst A, Andersen GL, Knight R, Hugenholtz P. 2012. An improved Greengenes taxonomy with explicit ranks for ecological and evolutionary analyses of bacteria and archaea. ISME J 6:610–618. 10.1038/ismej.2011.139 22134646 PMC3280142

[76] DeSantis TZ, Hugenholtz P, Larsen N, Rojas M, Brodie EL, Keller K, Huber T, Dalevi D, Hu P, Andersen GL. 2006. Greengenes, a chimera-checked 16S rRNA gene database and workbench compatible with ARB. Appl Environ Microbiol 72:5069–5072. 10.1128/AEM.03006-05 16820507 PMC1489311

[77] Du J, Khemmani M, Halverson T, Ene A, Limeira R, Tinawi L, Hochstedler-Kramer BR, Noronha MF, Putonti C, Wolfe AJ. 2024. Cataloging the phylogenetic diversity of human bladder bacterial isolates. Genome Biol 25:75. 10.1186/s13059-024-03216-8 38515176 PMC10958879

[78] Komesu YM, Dinwiddie DL, Richter HE, Lukacz ES, Sung VW, Siddiqui NY, Zyczynski HM, Ridgeway B, Rogers RG, Arya LA, Mazloomdoost D, Levy J, Carper B, Gantz MG, Eunice Kennedy Shriver National Institute of Child Health and Human Development Pelvic Floor Disorders Network. 2020. Defining the relationship between vaginal and urinary microbiomes. Am J Obstet Gynecol 222:154. 10.1016/j.ajog.2019.08.011 PMC6995424 31421123

[79] Biehl LM, Farowski F, Hilpert C, Nowag A, Kretzschmar A, Jazmati N, Tsakmaklis A, Wieters I, Khodamoradi Y, Wisplinghoff H, Vehreschild MJGT. 2022. Longitudinal variability in the urinary microbiota of healthy premenopausal women and the relation to neighboring microbial communities: a pilot study. PLoS One 17:e0262095. 10.1371/journal.pone.0262095 35030190 PMC8759677

[80] Zou L, Zhang Z, Chen J, Guo R, Tong X, Ju Y, Lu H, Yang H, Wang J, Zong Y, Xu X, Jin X, Xiao L, Jia H, Zhang T, Liu X. 2024. Unraveling the impact of host genetics and factors on the urinary microbiome in a young population. mBio 15:e0277324. 10.1128/mbio.02773-24 39513726 PMC11633168

[81] Bi Y, Wang Y, Li W, Chen Y, Qin J, Zheng H. 2025. Microbiota analysis of perimenopausal women experiencing recurrent vaginitis in conjunction with urinary tract infection. BMC Microbiol 25:1. 10.1186/s12866-024-03709-3 39755613 PMC11699749

[82] Sung J, Larsen P, Halverson TM, Waters TP, Goodman JR, Wolfe AJ. 2024. First trimester “clean catch” urine and vaginal swab sample distinct microbiological niches. Microbiol Spectr 12:e0263823. 10.1128/spectrum.02638-23 38088549 PMC10782990

[83] Atkins H, Sabharwal B, Boger L, Stegman N, Kula A, Wolfe AJ, Banerjee S, Putonti C. 2024. Evidence of *Lactobacillus* strains shared between the female urinary and vaginal microbiota. Microb Genom 10:001267. 10.1099/mgen.0.001267 38949867 PMC11316553

[84] Chen YB, Hochstedler B, Pham TT, Acevedo-Alvarez M, Mueller ER, Wolfe AJ. 2020. The urethral microbiota: a missing link in the female urinary microbiota. J Urol 204:303–309. 10.1097/JU.0000000000000910 32118507

[85] Marrie TJ, Swantee CA, Hartlen M. 1980. Aerobic and anaerobic urethral flora of healthy females in various physiological age groups and of females with urinary tract infections. J Clin Microbiol 11:654–659. 10.1128/jcm.11.6.654-659.1980 7000816 PMC273480

[86] Fredsgaard L, Thorsteinsson K, Bundgaard-Nielsen C, Ammitzbøll N, Leutscher P, Chai Q, Jensen AM, Sørensen S, Pedersen LM, Hagstrøm S, Arenholt LTS. 2021. Description of the voided urinary microbiota in asymptomatic prepubertal children – A pilot study. J Pediatr Urol 17:545. 10.1016/j.jpurol.2021.03.019 34053859

[87] Zheng Z, Hu J, Li W, Ma K, Zhang C, Li K, Yao Y. 2023. Integrated microbiome and metabolome analysis reveals novel urinary microenvironmental signatures in interstitial cystitis/bladder pain syndrome patients. J Transl Med 21:266. 10.1186/s12967-023-04115-5 37076836 PMC10114403

[88] Lee H-Y, Wang J-W, Juan Y-S, Li C-C, Liu C-J, Cho SY, Yeh H-C, Chueh K-S, Wu W-J, Wu D-C. 2021. The impact of urine microbiota in patients with lower urinary tract symptoms. Ann Clin Microbiol Antimicrob 20:23. 10.1186/s12941-021-00428-9 33858430 PMC8051042

[89] Bowie KR, Garzotto M, Orwoll E, Karstens L. 2023. BMI and BPH correlate with urinary microbiome diversity and lower urinary tract symptoms in men. bioRxiv:2023.12.14.571758. 10.1101/2023.12.14.571758 PMC12056106 40328908

[90] Mariotti ACH, Heidrich V, Inoue LT, Coser EM, dos Santos EX, dos Santos HDB, Rocha CBT, Asprino PF, Bettoni F, Bastos DA, Jardim DLF, Camargo AA, Arap MA. 2024. Urinary microbiota is associated to clinicopathological features in benign prostatic hyperplasia. Prostate 84:285–291. 10.1002/pros.24649 37961000

[91] Liu F, Zhang N, Wu Y, Jiang P, Jiang T, Wang Y, Zhang Y, Zhai Q, Zou Y, Feng N. 2020. The pelvis urinary microbiome in patients with kidney stones and clinical associations. BMC Microbiol 20:336. 10.1186/s12866-020-01992-4 33153435 PMC7643416

[92] Komesu YM, Richter HE, Carper B, Dinwiddie DL, Lukacz ES, Siddiqui NY, Sung VW, Zyczynski HM, Ridgeway B, Rogers RG, Arya LA, Mazloomdoost D, Gantz MG, Pelvic Floor Disorders Network. 2018. The urinary microbiome in women with mixed urinary incontinence compared to similarly aged controls. Int Urogynecol J 29:1785–1795. 10.1007/s00192-018-3683-6 29909556 PMC6295358

[93] Lillemon JN, Karstens L, Nardos R, Garg B, Boniface ER, Gregory WT. 2022. The impact of local estrogen on the urogenital microbiome in genitourinary syndrome of menopause: a randomized-controlled trial. Female Pelvic Med Reconstr Surg 28:e157–e162. 10.1097/SPV.0000000000001170 35420551

[94] Siddiqui NY, Ma L, Brubaker L, Mao J, Hoffman C, Dahl EM, Wang Z, Karstens L. 2022. Updating urinary microbiome analyses to enhance biologic interpretation. Front Cell Infect Microbiol 12:789439. 10.3389/fcimb.2022.789439 35899056 PMC9309214

[95] Dalvi H, De Nisco NJ. 2024. The evolving world of the urinary microbiome. Curr Opin Urol 34:422–427. 10.1097/MOU.0000000000001222 39224916

[96] Timm MR, Russell SK, Hultgren SJ. 2025. Urinary tract infections: pathogenesis, host susceptibility and emerging therapeutics. Nat Rev Microbiol 23:72–86. 10.1038/s41579-024-01092-4 39251839 PMC13194463

[97] Flores-Mireles AL, Walker JN, Caparon M, Hultgren SJ. 2015. Urinary tract infections: epidemiology, mechanisms of infection and treatment options. Nat Rev Microbiol 13:269–284. 10.1038/nrmicro3432 25853778 PMC4457377

[98] Gaston JR, Johnson AO, Bair KL, White AN, Armbruster CE. 2021. Polymicrobial interactions in the urinary tract: is the enemy of my enemy my friend? Infect Immun:IAI.00652–20. 10.1128/IAI.00652-20 33431702

[99] Kline KA, Lewis AL. 2016. Gram-positive uropathogens, polymicrobial urinary tract infection, and the emerging microbiota of the urinary tract. Microbiol Spectr 4. 10.1128/microbiolspec.UTI-0012-2012 PMC4888879 27227294

[100] Ravel J, Gajer P, Abdo Z, Schneider GM, Koenig SSK, McCulle SL, Karlebach S, Gorle R, Russell J, Tacket CO, Brotman RM, Davis CC, Ault K, Peralta L, Forney LJ. 2011. Vaginal microbiome of reproductive-age women. Proc Natl Acad Sci USA 108:4680–4687. 10.1073/pnas.1002611107 20534435 PMC3063603

[101] Arumugam M, Raes J, Pelletier E, Le Paslier D, Yamada T, Mende DR, Fernandes GR, Tap J, Bruls T, Batto J-M, 2011. Enterotypes of the human gut microbiome. Nature 473:174–180. 10.1038/nature09944 21508958 PMC3728647

[102] Costea PI, Hildebrand F, Arumugam M, Bäckhed F, Blaser MJ, Bushman FD, de Vos WM, Ehrlich SD, Fraser CM, Hattori M, 2018. Enterotypes in the landscape of gut microbial community composition. Nat Microbiol 3:8–16. 10.1038/s41564-017-0072-8 29255284 PMC5832044

[103] Pearce MM, Zilliox MJ, Rosenfeld AB, Thomas-White KJ, Richter HE, Nager CW, Visco AG, Nygaard IE, Barber MD, Schaffer J, Moalli P, Sung VW, Smith AL, Rogers R, Nolen TL, Wallace D, Meikle SF, Gai X, Wolfe AJ, Brubaker L, Pelvic Floor Disorders Network. 2015. The female urinary microbiome in urgency urinary incontinence. Am J Obstet Gynecol 213:347. 10.1016/j.ajog.2015.07.009 PMC4556587 26210757

[104] Song CH, Kim YH, Naskar M, Hayes BW, Abraham MA, Noh JH, Suk G, Kim MJ, Cho KS, Shin M, Lee E-J, Abraham SN, Choi HW. 2022. *Lactobacillus crispatus* limits bladder uropathogenic *E. coli* infection by triggering a host type I interferon response. Proc Natl Acad Sci USA 119:e2117904119. 10.1073/pnas.2117904119 35939684 PMC9388105

[105] Johnson JA, Delaney LF, Ojha V, Rudraraju M, Hintze KR, Siddiqui NY, Sysoeva TA. 2022. Commensal urinary lactobacilli inhibit major uropathogens *in vitro* with heterogeneity at species and strain level. Front Cell Infect Microbiol 12:870603. 10.3389/fcimb.2022.870603 35811675 PMC9260849

[106] Perez RH, Zendo T, Sonomoto K. 2014. Novel bacteriocins from lactic acid bacteria (LAB): various structures and applications. Microb Cell Fact 13:S3. 10.1186/1475-2859-13-S1-S3 25186038 PMC4155820

[107] Karlsson M, Scherbak N, Reid G, Jass J. 2012. *Lactobacillus rhamnosus* GR-1 enhances NF-kappaB activation in *Escherichia coli*-stimulated urinary bladder cells through TLR4. BMC Microbiol 12:15. 10.1186/1471-2180-12-15 22264349 PMC3305351

[108] Pelayo P, Hussain FA, Werlang CA, Wu CM, Woolston BM, Xiang CM, Rutt L, France MT, Ravel J, Ribbeck K, Kwon DS, Balskus EP. 2024. *Prevotella* are major contributors of sialidases in the human vaginal microbiome. Proc Natl Acad Sci USA 121:e2400341121. 10.1073/pnas.2400341121 39186657 PMC11388281

[109] Segui-Perez C, de Jongh R, Jonkergouw RLW, Pelayo P, Balskus EP, Zomer A, Strijbis K. 2024. Prevotella timonensis degrades the vaginal epithelial glycocalyx through high fucosidase and sialidase activities. mBio 15:e0069124. 10.1128/mbio.00691-24 39162399 PMC11389373

[110] Precup G, Vodnar DC. 2019. Gut *Prevotella* as a possible biomarker of diet and its eubiotic versus dysbiotic roles: a comprehensive literature review. Br J Nutr 122:131–140. 10.1017/S0007114519000680 30924428

[111] Lotte L, Lotte R, Durand M, Degand N, Ambrosetti D, Michiels J-F, Amiel J, Cattoir V, Ruimy R. 2016. Infections related to Actinotignum schaalii (formerly Actinobaculum schaalii): a 3-year prospective observational study on 50 cases. Clin Microbiol Infect 22:388–390. 10.1016/j.cmi.2015.10.030 26551841

[112] Yassin AF, Spröer C, Pukall R, Sylvester M, Siering C, Schumann P. 2015. Dissection of the genus Actinobaculum: reclassification of Actinobaculum schaalii Lawson et al. 1997 and Actinobaculum urinale Hall et al. 2003 as Actinotignum schaalii gen. nov., comb. nov. and Actinotignum urinale comb. nov., description of Actinotignum sanguinis sp. nov. and emended descriptions of the genus Actinobaculum and Actinobaculum suis; and re-examination of the culture deposited as Actinobaculum massiliense CCUG 47753T ( = DSM 19118T), revealing that it does not represent a strain of this species. Int J Syst Evol Microbiol 65:615–624. 10.1099/ijs.0.069294-0 25406238

[113] Lawson PA, Falsen E, Akervall E, Vandamme P, Collins MD. 1997. Characterization of some Actinomyces-like isolates from human clinical specimens: reclassification of Actinomyces suis (Soltys and Spratling) as Actinobaculum suis comb. nov. and description of Actinobaculum schaalii sp. nov. Int J Syst Bacteriol 47:899–903. 10.1099/00207713-47-3-899 9226926

[114] Hall V, Collins MD, Hutson RA, Falsen E, Inganäs E, Duerden BI. 2003. *Actinobaculum urinale* sp. nov., from human urine. Int J Syst Evol Microbiol 53:679–682. 10.1099/ijs.0.02422-0 12807186

[115] Panganiban CM, Gupta S. 2020. *Actinotignum schaalii* abscess in a patient with common variable immunodeficiency. Pathogens 9:494. 10.3390/pathogens9060494 32580346 PMC7350303

[116] Washio M, Harada N, Nishima D, Takemoto M. 2022. Actinotignum schaalii can be an uropathogen of “culture-negative” febrile urinary tract infections in children with urinary tract abnormalities. Case Rep Nephrol Dial 12:150–156. 10.1159/000526398 36518361 PMC9743144

[117] Lotte L, Durand C, Chevalier A, Gaudart A, Cheddadi Y, Ruimy R, Lotte R. 2023. Acute pyelonephritis with bacteremia in an 89-year-old woman caused by two slow-growing bacteria: *Aerococcus urinae* and *Actinotignum schaalii*. Microorganisms 11:2908. 10.3390/microorganisms11122908 38138052 PMC10746031

[118] Wang D, Haley E, Luke N, Mathur M, Festa RA, Zhao X, Anderson LA, Allison JL, Stebbins KL, Diaz MJ, Baunoch D. 2023. Emerging and fastidious uropathogens were detected by M-PCR with similar prevalence and cell density in catheter and midstream voided urine indicating the importance of these microbes in causing UTIs. Infect Drug Resist 16:7775–7795. 10.2147/IDR.S429990 38148772 PMC10750486

[119] Beguelin C, Genne D, Varca A, Tritten M-L, Siegrist HH, Jaton K, Lienhard R. 2011. *Actinobaculum schaalii*: clinical observation of 20 cases. Clin Microbiol Infect 17:1027–1031. 10.1111/j.1469-0691.2010.03370.x 20854424

[120] Lieu A, Mah J, Peirano G, Somayaji R, Church D. 2022. Microbiological characterization of *Actinotignum schaalii* strains causing invasive infections during a multiyear period in a large Canadian health care region. Microbiol Spectr 10:e0344222. 10.1128/spectrum.03442-22 36409090 PMC9769901

[121] Horton LE, Mehta SR, Aganovic L, Fierer J. 2018. Actinotignum schaalii infection: a clandestine cause of sterile pyuria? Open Forum Infect Dis 5:ofy015. 10.1093/ofid/ofy015 29450211 PMC5808804

[122] Sahuquillo-Arce JM, Suárez-Urquiza P, Hernández-Cabezas A, Tofan L, Chouman-Arcas R, García-Hita M, Sabalza-Baztán O, Sellés-Sánchez A, Lozano-Rodríguez N, Martí-Cuñat J, López-Hontangas JL. 2024. *Actinotignum schaalii* infection: challenges in diagnosis and treatment. Heliyon 10:e28589. 10.1016/j.heliyon.2024.e28589 38590897 PMC10999919

[123] Yassin AF, Langenberg S, Huntemann M, Clum A, Pillay M, Palaniappan K, Varghese N, Mikhailova N, Mukherjee S, Reddy TBK, Daum C, Shapiro N, Ivanova N, Woyke T, Kyrpides NC. 2017. Draft genome sequence of *Actinotignum schaalii* DSM 15541T: genetic insights into the lifestyle, cell fitness and virulence. PLoS One 12:e0188914. 10.1371/journal.pone.0188914 29216246 PMC5720513

[124] Higgins A, Garg T. 2017. *Aerococcus urinae*: an emerging cause of urinary tract infection in older adults with multimorbidity and urologic cancer. Urol Case Rep 13:24–25. 10.1016/j.eucr.2017.03.022 28435789 PMC5393163

[125] Rast D, Evers KS, Egli A, Rudin C, Goischke A, Ritz N. 2023. *Aerococcus urinae* — significance of detection in the paediatric urinary tract: a case series. Eur J Pediatr 182:749–756. 10.1007/s00431-022-04730-2 36472648 PMC9899180

[126] Hilt EE, Putonti C, Thomas-White K, Lewis AL, Visick KL, Gilbert NM, Wolfe AJ. 2020. *Aerococcus urinae* isolated from women with lower urinary tract symptoms: *in vitro* aggregation and genome analysis. J Bacteriol 202:e00170–20. 10.1128/JB.00170-20 32284319 PMC7283593

[127] Yu Y, Tsitrin T, Bekele S, Thovarai V, Torralba MG, Singh H, Wolcott R, Doerfert SN, Sizova MV, Epstein SS, Pieper R. 2019. *Aerococcus urinae* and *Globicatella sanguinis* persist in polymicrobial urethral catheter biofilms examined in longitudinal profiles at the proteomic level. Biochem Insights 12:1178626419875089. 10.1177/1178626419875089 31555049 PMC6753514

[128] Nye TM, Zou Z, Obernuefemann CLP, Pinkner JS, Lowry E, Kleinschmidt K, Bergeron K, Klim A, Dodson KW, Flores-Mireles AL, Walker JN, Wong DG, Desai A, Caparon MG, Hultgren SJ. 2024. Microbial co-occurrences on catheters from long-term catheterized patients. Nat Commun 15:61. 10.1038/s41467-023-44095-0 38168042 PMC10762172

[129] Gilbert NM, Choi B, Du J, Collins C, Lewis AL, Putonti C, Wolfe AJ. 2021. A mouse model displays host and bacterial strain differences in Aerococcus urinae urinary tract infection. Biol Open 10:bio058931. 10.1242/bio.058931 34387311 PMC8380466

[130] Choi BI, Ene A, Du J, Johnson G, Putonti C, Schouw CH, Dargis R, Senneby E, Christensen JJ, Wolfe AJ. 2023. Taxonomic considerations on *Aerococcus urinae* with proposal of subdivision into *Aerococcus urinae*, *Aerococcus tenax* sp. nov., *Aerococcus mictus* sp. nov., and *Aerococcus loyolae* sp. nov. Int J Syst Evol Microbiol 73. 10.1099/ijsem.0.006066 37755156

[131] Devlin CM, Simms MS, Maitland NJ. 2021. Benign prostatic hyperplasia – what do we know? BJU Int 127:389–399. 10.1111/bju.15229 32893964

[132] Tschudin-Sutter S, Frei R, Weisser M, Goldenberger D, Widmer AF. 2011. *Actinobaculum schaalii* - invasive pathogen or innocent bystander? A retrospective observational study. BMC Infect Dis 11:289. 10.1186/1471-2334-11-289 22029906 PMC3252262

[133] Porto JG, Arbelaez MCS, Pena B, Khandekar A, Malpani A, Nahar B, Punnen S, Ritch CR, Gonzalgo ML, Parekh DJ, Marcovich R, Shah HN. 2023. The influence of the microbiome on urological malignancies: a systematic review. Cancers (Basel) 15:4984. 10.3390/cancers15204984 37894351 PMC10605095

[134] Chen VS, James C, Khemmani M, Desai S, Doshi C, Rac G, Ellis JL, Patel HD, Barkan GA, Gupta GN, Flanigan RC, Wolfe AJ. 2024. A prospective evaluation of the prostate microbiome in malignant and benign tissue using transperineal biopsy. Prostate 84:1251–1261. 10.1002/pros.24763 38946139

[135] Anger JT, Dallas KB, Bresee C, De Hoedt AM, Barbour KE, Hoggatt KJ, Goodman MT, Kim J, Freedland SJ. 2022. National prevalence of IC/BPS in women and men utilizing veterans health administration data. Front Pain Res (Lausanne) 3:925834. 10.3389/fpain.2022.925834 36093391 PMC9448885

[136] Suskind AM, Berry SH, Ewing BA, Elliott MN, Suttorp MJ, Clemens JQ. 2013. The prevalence and overlap of interstitial cystitis/bladder pain syndrome and chronic prostatitis/chronic pelvic pain syndrome in men: results of the rand interstitial cystitis epidemiology male study. J Urol 189:141–145. 10.1016/j.juro.2012.08.088 23164386 PMC3894747

[137] Berry SH, Bogart LM, Pham C, Liu K, Nyberg L, Stoto M, Suttorp M, Clemens JQ. 2010. Development, validation and testing of an epidemiological case definition of interstitial cystitis/painful bladder syndrome. J Urol 183:1848–1852. 10.1016/j.juro.2009.12.103 20303099 PMC3519367

[138] Ueda T, Hanno PM, Saito R, Meijlink JM, Yoshimura N. 2021. Current understanding and future perspectives of interstitial cystitis/bladder pain syndrome. Int Neurourol J 25:99–110. 10.5213/inj.2142084.042 34218637 PMC8255826

[139] Haarala M, Kiilholma P, Lehtonen OP. 1999. Urinary bacterial flora of women with urethral syndrome and interstitial cystitis. Gynecol Obstet Invest 47:42–44. 10.1159/000010060 9852391

[140] Heritz DM, Lacroix J-M, Batra SD, Jarvi KA, Beheshti B, Mittelman MW. 1997. Detection of eubacteria in interstitial cystitis by 16S rDNA amplification. J Urol 158:2291–2295. 10.1016/s0022-5347(01)68237-5 9366378

[141] Haarala M, Jalava J, Laato M, Kiilholma P, Nurmi M, Alanen A. 1996. Absence of bacterial DNA in the bladder of patients with interstitial cystitis. J Urol 156:1843–1845. 10.1016/S0022-5347(01)65549-6 8863628

[142] Al-Hadithi HN, Williams H, Hart CA, Frazer M, Adams EJ, Richmond DH, Tincello DG. 2005. Absence of bacterial and viral DNA in bladder biopsies from patients with interstitial cystitis/chronic pelvic pain syndrome. J Urol 174:151–154. 10.1097/01.ju.0000161605.14804.a9 15947607

[143] Hampson SJ, Christmas TJ, Moss MT. 1993. Search for mycobacteria in interstitial cystitis using mycobacteria-specific DNA probes with signal amplication by polymerase chain reaction. Br J Urol 72:303–306. 10.1111/j.1464-410X.1993.tb00722.x 8220991

[144] Agarwal M, Dixon RA. 2001. A study to detect *Gardnerella vaginalis* DNA in interstitial cystitis. BJU Int 88:868–870. 10.1046/j.1464-4096.2001.01441.x 11851605

[145] Nickel JC, Ehrlich GD, Krol JE, Ahmed A, Sen B, Bhat A, Mell JC, Doiron RC, Kelly K-L, Earl JP. 2022. The bacterial microbiota of Hunner lesion interstitial cystitis/bladder pain syndrome. BJU Int 129:104–112. 10.1111/bju.15519 34143561

[146] Domingue GJ, Ghoniem GM, Bost KL, Fermin C, Human LG. 1995. Dormant microbes in interstitial cystitis. J Urol 153:1321–1326. 10.1016/S0022-5347(01)67594-3 7869536

[147] Abernethy MG, Rosenfeld A, White JR, Mueller MG, Lewicky-Gaupp C, Kenton K. 2017. Urinary microbiome and cytokine levels in women with interstitial cystitis. Obstet Gynecol 129:500–506. 10.1097/AOG.0000000000001892 28178051

[148] Xu H, Tamrat NE, Gao J, Xu J, Zhou Y, Zhang S, Chen Z, Shao Y, Ding L, Shen B, Wei Z. 2021. Combined signature of the urinary microbiome and metabolome in patients with interstitial cystitis. Front Cell Infect Microbiol 11:711746. 10.3389/fcimb.2021.711746 34527602 PMC8436771

[149] Walton I, Nickel JC. 2021. The urinary microbiome in interstitial cystitis/bladder pain syndrome? A critical look at the evidence. J Urol 206:1087–1090. 10.1097/JU.0000000000001947 34184928

[150] Hashemi N, Tondro Anamag F, Javan Balegh Marand A, Rahnama’i MS, Herizchi Ghadim H, Salehi-Pourmehr H, Hajebrahimi S. 2024. A systematic and comprehensive review of the role of microbiota in urinary chronic pelvic pain syndrome. Neurourol Urodyn 43:1859–1882. 10.1002/nau.25550 38994675

[151] Fu C, Zhang Y, Liang L, Lin H, Shan K, Liu F, Feng N. 2024. The microbiota in patients with interstitial cystitis/bladder pain syndrome: a systematic review. BJU Int 134:869–880. 10.1111/bju.16439 38890150

[152] Nickel JC, Stephens-Shields AJ, Landis JR, Mullins C, van Bokhoven A, Lucia MS, Henderson JP, Sen B, Krol JE, Ehrlich GD, MAPP Research Network. 2019. A culture-independent analysis of the microbiota of female interstitial cystitis/bladder pain syndrome participants in the MAPP research network. J Clin Med 8:415. 10.3390/jcm8030415 30917614 PMC6462969

[153] Bresler L, Price TK, Hilt EE, Joyce C, Fitzgerald CM, Wolfe AJ. 2019. Female lower urinary tract microbiota do not associate with IC/PBS symptoms: a case-controlled study. Int Urogynecol J 30:1835–1842. 10.1007/s00192-019-03942-9 30993388

[154] Nickel JC, Stephens A, Landis JR, Chen J, Mullins C, van Bokhoven A, Lucia MS, Melton-Kreft R, Ehrlich GD, MAPP Research Network. 2015. Search for microorganisms in men with urologic chronic pelvic pain syndrome: a culture-independent analysis in the MAPP research network. J Urol 194:127–135. 10.1016/j.juro.2015.01.037 25596358 PMC4475477

[155] Nickel JC, Stephens A, Landis JR, Mullins C, van Bokhoven A, Lucia MS, Ehrlich GD, MAPP Research Network. 2016. Assessment of the lower urinary tract microbiota during symptom flare in women with urologic chronic pelvic pain syndrome: a MAPP network study. J Urol 195:356–362. 10.1016/j.juro.2015.09.075 26410734 PMC4770794

[156] Duncan JL, Schaeffer AJ. 1997. Do infectious agents cause interstitial cystitis? Urology 49:48–51. 10.1016/s0090-4295(99)80331-8 9146001

[157] Abernethy MG, Tsuei A. 2021. The bladder microbiome and interstitial cystitis: is there a connection? Curr Opin Obstet Gynecol 33:469–473. 10.1097/GCO.0000000000000747 34475365

[158] Parsons CL. 2015. How does interstitial cystitis begin? Transl Androl Urol 4:605–610. 10.3978/j.issn.2223-4683.2015.11.02 26816860 PMC4708543

[159] Warren JW. 1994. Is interstitial cystitis an infectious disease? Med Hypotheses 43:183–186. 10.1016/0306-9877(94)90150-3 7815976

[160] Clemens JQ, Erickson DR, Varela NP, Lai HH. 2022. Diagnosis and treatment of interstitial cystitis/bladder pain syndrome. J Urol 208:34–42. 10.1097/JU.0000000000002756 35536143

[161] Li K, Chen C, Zeng J, Wen Y, Chen W, Zhao J, Wu P. 2022. Interplay between bladder microbiota and overactive bladder symptom severity: a cross-sectional study. BMC Urol 22:39. 10.1186/s12894-022-00990-0 35305613 PMC8934487

[162] Brubaker L, Nager CW, Richter HE, Visco A, Nygaard I, Barber MD, Schaffer J, Meikle S, Wallace D, Shibata N, Wolfe AJ. 2014. Urinary bacteria in adult women with urgency urinary incontinence. Int Urogynecol J 25:1179–1184. 10.1007/s00192-013-2325-2 24515544 PMC4128900

[163] Thomas-White KJ, Hilt EE, Fok C, Pearce MM, Mueller ER, Kliethermes S, Jacobs K, Zilliox MJ, Brincat C, Price TK, Kuffel G, Schreckenberger P, Gai X, Brubaker L, Wolfe AJ. 2016. Incontinence medication response relates to the female urinary microbiota. Int Urogynecol J 27:723–733. 10.1007/s00192-015-2847-x 26423260 PMC5119460

[164] Thomas-White KJ, Kliethermes S, Rickey L, Lukacz ES, Richter HE, Moalli P, Zimmern P, Norton P, Kusek JW, Wolfe AJ, Brubaker L, National Institute of Diabetes and Digestive and Kidney Diseases Urinary Incontinence Treatment Network. 2017. Evaluation of the urinary microbiota of women with uncomplicated stress urinary incontinence. Am J Obstet Gynecol 216:55. 10.1016/j.ajog.2016.07.049 PMC5182144 27498309

[165] Nardos R, Leung ET, Dahl EM, Davin S, Asquith M, Gregory WT, Karstens L. 2022. Network-based differences in the vaginal and bladder microbial communities between women with and without urgency urinary incontinence. Front Cell Infect Microbiol 12:759156. 10.3389/fcimb.2022.759156 35402312 PMC8988226

[166] Abbasian B, Shair A, O’Gorman DB, Pena-Diaz AM, Brennan L, Engelbrecht K, Koenig DW, Reid G, Burton JP. 2019. Potential role of extracellular ATP released by bacteria in bladder infection and contractility. mSphere 4:e00439–19. 10.1128/mSphere.00439-19 31484739 PMC6731529

[167] Pearle MS, Goldfarb DS, Assimos DG, Curhan G, Denu-Ciocca CJ, Matlaga BR, Monga M, Penniston KL, Preminger GM, Turk TMT, White JR, American Urological Assocation. 2014. Medical management of kidney stones: AUA guideline. J Urol 192:316–324. 10.1016/j.juro.2014.05.006 24857648

[168] Zampini A, Nguyen AH, Rose E, Monga M, Miller AW. 2019. Defining dysbiosis in patients with urolithiasis. Sci Rep 9:5425. 10.1038/s41598-019-41977-6 30932002 PMC6443657

[169] Kachroo N, Monga M, Miller AW. 2022. Comparative functional analysis of the urinary tract microbiome for individuals with or without calcium oxalate calculi. Urolithiasis 50:303–317. 10.1007/s00240-022-01314-5 35234986 PMC11247624

[170] Noonin C, Putpim A, Thongboonkerd V. 2024. The direct inhibitory effects of Lactobacillus acidophilus, a commensal urinary bacterium, on calcium oxalate stone development. Microbiome 12:175. 10.1186/s40168-024-01877-y 39289694 PMC11406782

[171] Barr-Beare E, Saxena V, Hilt EE, Thomas-White K, Schober M, Li B, Becknell B, Hains DS, Wolfe AJ, Schwaderer AL. 2015. The interaction between Enterobacteriaceae and calcium oxalate deposits. PLoS One 10:e0139575. 10.1371/journal.pone.0139575 26448465 PMC4598009

[172] Lemberger U, Pjevac P, Hausmann B, Berry D, Moser D, Jahrreis V, Özsoy M, Shariat SF, Veser J. 2023. The microbiome of kidney stones and urine of patients with nephrolithiasis. Urolithiasis 51:27. 10.1007/s00240-022-01403-5 36596939 PMC9810570

[173] Bichler K-H, Eipper E, Naber K, Braun V, Zimmermann R, Lahme S. 2002. Urinary infection stones. Int J Antimicrob Agents 19:488–498. 10.1016/s0924-8579(02)00088-2 12135839

[174] Shen C, Zhu Q, Dong F, Wang W, Fan B, Li K, Chen J, Hu S, He Z, Li X. 2021. Identifying two novel clusters in calcium oxalate stones with urinary tract infection using 16S rDNA sequencing. Front Cell Infect Microbiol 11:723781. 10.3389/fcimb.2021.723781 34869053 PMC8635737

[175] Soriano F, Ponte C, Santamaría M, Castilla C, Fernández Roblas R. 1986. *In vitro* and *in vivo* study of stone formation by *Corynebacterium* group D2 (Corynebacterium urealyticum). J Clin Microbiol 23:691–694. 10.1128/jcm.23.4.691-694.1986 3517059 PMC362818

[176] Bermudez T, Schmitz JE, Boswell M, Humphries R. 2025. Novel technologies for the diagnosis of urinary tract infections. J Clin Microbiol 63:e0030624. 10.1128/jcm.00306-24 39760497 PMC11837515

[177] Grin PM, Kowalewska PM, Alhazzan W, Fox-Robichaud AE. 2013. *Lactobacillus* for preventing recurrent urinary tract infections in women: meta-analysis. Can J Urol 20:6607–6614. 23433130

[178] Falagas ME, Betsi GI, Tokas T, Athanasiou S. 2006. Probiotics for prevention of recurrent urinary tract infections in women: a review of the evidence from microbiological and clinical studies. Drugs (Abingdon Engl) 66:1253–1261. 10.2165/00003495-200666090-00007 16827601

[179] Filkins LM, Graber JA, Olson DG, Dolben EL, Lynd LR, Bhuju S, O’Toole GA. 2015. Coculture of *Staphylococcus aureus* with *Pseudomonas aeruginosa* drives *S. aureus* towards fermentative metabolism and reduced viability in a cystic fibrosis model. J Bacteriol 197:2252–2264. 10.1128/JB.00059-15 25917910 PMC4524177

[180] Jean-Pierre F, Hampton TH, Schultz D, Hogan DA, Groleau M-C, Déziel E, O’Toole GA. 2023. Community composition shapes microbial-specific phenotypes in a cystic fibrosis polymicrobial model system. Elife 12:e81604. 10.7554/eLife.81604 36661299 PMC9897730

[181] Samuel BS, Gordon JI. 2006. A humanized gnotobiotic mouse model of host-archaeal-bacterial mutualism. Proc Natl Acad Sci USA 103:10011–10016. 10.1073/pnas.0602187103 16782812 PMC1479766

[182] Brooks T, Keevil CW. 1997. A simple artificial urine for the growth of urinary pathogens. Lett Appl Microbiol 24:203–206. 10.1046/j.1472-765x.1997.00378.x 9080700

[183] Sarigul N, Korkmaz F, Kurultak İ. 2019. A new artificial urine protocol to better imitate human urine. Sci Rep 9:20159. 10.1038/s41598-019-56693-4 31882896 PMC6934465

[184] Griffith DP, Musher DM, Itin C. 1976. Urease. The primary cause of infection-induced urinary stones. Invest Urol 13:346–350. 815197

[185] Stickler DJ, Morris NS, Winters C. 1999. Simple physical model to study formation and physiology of biofilms on urethral catheters. Methods Enzymol 310:494–501. 10.1016/s0076-6879(99)10037-5 10547813

[186] Neugent ML, Hulyalkar NV, Kumar A, Xing C, Zimmern PE, Shulaev V, De Nisco NJ. 2023. Urinary glycosaminoglycans are associated with recurrent UTI and urobiome ecology in postmenopausal women. ACS Infect Dis 9:1022–1032. 10.1021/acsinfecdis.3c00027 36942838 PMC10111421

